# Global, regional, and national burden of spinal cord injury, 1990–2019: a systematic analysis for the Global Burden of Disease Study 2019

**DOI:** 10.1016/S1474-4422(23)00287-9

**Published:** 2023-11

**Authors:** GBD Spinal Cord Injuries Collaborators, Mahdi Safdarian, Eugen Trinka, Vafa Rahimi-Movaghar, Aljoscha Thomschewski, Amirali Aali, Gdiom Gebreheat Abady, Semagn Mekonnen Abate, Foad Abd-Allah, Aidin Abedi, Denberu Eshetie Adane, Saira Afzal, Bright Opoku Ahinkorah, Sajjad Ahmad, Haroon Ahmed, Nasir Amanat, Dhanalakshmi Angappan, Jalal Arabloo, Armin Aryannejad, Seyyed Shamsadin Athari, Alok Atreya, Sina Azadnajafabad, Ahmed Y Azzam, Hassan Babamohamadi, Palash Chandra Banik, Mainak Bardhan, Azadeh Bashiri, Alemshet Yirga Berhie, Ajay Nagesh Bhat, Julie Brown, Ana Paula Champs, Periklis Charalampous, Isaac Sunday Chukwu, Kaleb Coberly, Omid Dadras, Dereje Y Yada, Xiaochen Dai, Lalit Dandona, Rakhi Dandona, Fikadu Nugusu Dessalegn, Abebaw Alemayehu Desta, Sameer Dhingra, Nancy Diao, Daniel Diaz, Mahmoud Dibas, Deepa Dongarwar, Haneil Larson Dsouza, Michael Ekholuenetale, Nevine El Nahas, Muhammed Elhadi, Sharareh Eskandarieh, Adeniyi Francis Fagbamigbe, Jawad Fares, Ali Fatehizadeh, Seyed-Mohammad Fereshtehnejad, Florian Fischer, Richard Charles Franklin, Tushar Garg, Melaku Getachew, Fariborz Ghaffarpasand, Ali Gholamrezanezhad, Milad Gholizadeh Mesgarha, Sherief Ghozy, Mahaveer Golechha, Pouya Goleij, Simon Matthew Graham, Vivek Kumar Gupta, Juanita A. Haagsma, Samer Hamidi, Netanja I. Harlianto, Mehdi Harorani, Mohammad Hasanian, Amr Hassan, Mohammed Bheser Hassen, Amir Human Hoveidaei, Farideh Iravanpour, Rana Irilouzadian, Chidozie C D Iwu, Louis Jacob, Chinwe Juliana Jaja, Nitin Joseph, Charity Ehimwenma Joshua, Jacek Jerzy Jozwiak, Vidya Kadashetti, Amit Kandel, Rami S. Kantar, Ibraheem M Karaye, Samad Karkhah, Yousef Saleh Khader, Ejaz Ahmad Khan, Md Jobair Khan, Hamid Reza Khayat Kashani, Mohammad Saeid Khonji, Moein Khormali, Grace Kim, Vijay Krishnamoorthy, Senthil D Kumaran, Mohammad-Reza Malekpour, Tuomo J Meretoja, Mohamed Kamal Mesregah, Tomislav Mestrovic, Ana Carolina Micheletti Gomide Nogueira de Sá, Alireza Mirahmadi, Seyed Peyman Mirghaderi, Moonis Mirza, Awoke Misganaw, Sanjeev Misra, Yousef Mohammad, Esmaeil Mohammadi, Ali H Mokdad, Holger Möller, Sara Momtazmanesh, Mohammad Ali Moni, Ebrahim Mostafavi, Francesk Mulita, Mohsen Naghavi, Hasan Nassereldine, Zuhair S Natto, Kazem Nejati, Huong Lan Thi Nguyen, Van Thanh Nguyen, Antonio Tolentino Nogueira de Sá, Andrew T Olagunju, Isaac Iyinoluwa Olufadewa, Abiodun Olusola Omotayo, Mayowa O Owolabi, Shankargouda Patil, Shrikant Pawar, Paolo Pedersini, Ionela-Roxana Petcu, Suzanne Polinder, Ali Mohammad Pourbagher-Shahri, Maryam Faiz Qureshi, Pankaja Raghav Raghav, Mosiur Rahman, Niloufar Rahnavard, Ali Rajabpour-Sanati, Mohammad-Mahdi Rashidi, Salman Rawaf, Nicholas L S Roberts, Basema Saddik, Umar Saeed, Sara Samadzadeh, Abdallah M Samy, Arash Sarveazad, Allen Seylani, Mahan Shafie, Ataollah Shahbandi, Mequannent Melaku Sharew Sharew, Rahim Ali Sheikhi, Pavanchand H Shetty, Arzu Yigit, Parnian Shobeiri, Sina Shool, Seyed Afshin Shorofi, Migbar Mekonnen Sibhat, Ehsan Sinaei, Paramdeep Singh, Surjit Singh, Yonatan Solomon, Houman Sotoudeh, Belsti Atnkut Tadesse, Muhammad Umair, Sahel Valadan Tahbaz, Pascual R Valdez, Narayanaswamy Venketasubramanian, Linh Gia Vu, Nuwan Darshana Wickramasinghe, Iman Zare, Fereshteh Yazdanpanah, Ai-Min Wu, Zhi-Jiang Zhang

## Abstract

**Background:**

Spinal cord injury (SCI) is a major cause of health loss due to premature mortality and long-term disability. We aimed to report on the global, regional, and national incidence, prevalence, and years of life lived with disability (YLDs) for SCI from 1990 to 2019, using data from the Global Burden of Diseases, Injuries, and Risk Factors Study (GBD) 2019.

**Methods:**

Using GBD 2019 data pooled in DisMod-MR 2.1, a Bayesian meta-regression tool, we systematically derived numbers and age-standardised rate changes with 95% uncertainty intervals (95% UIs) for the incidence, prevalence, and YLDs for SCI from 1990 to 2019 for the whole world, 21 GBD regions, and 204 countries and territories. We report trends based on age, sex, year, cause of injury, and level of injury.

**Findings:**

Globally, 20·6 million (95% UI 18·9 to 23·6) individuals were living with SCI in 2019. The incidence of SCI was 0·9 million (0·7 to 1·2) cases with an estimated 6·2 million (4·5 to 8·2) YLDs. SCI rates increased substantially from 1990 to 2019 for global prevalence (81·5%, 74·2 to 87·1), incidence (52·7%, 30·3 to 69·8), and YLDs (65·4%, 56·3 to 76·0). However, global age-standardised rates per 100 000 population showed small changes in prevalence (5·8%, 2·6 to 9·5), incidence (–6·1%, –17·2 to 1·5), and YLDs (–1·5%, –5·5 to 3·2). Data for 2019 shows that the incidence of SCI increases sharply until age 15–19 years, where it remains reasonably constant until 85 years of age and older. By contrast, prevalence and YLDs showed similar patterns to each other, with one peak at around age 45–54 years. The incidence, prevalence, and YLDs of SCI have consistently been higher in men than in women globally, with a slight and steady increase for both men and women from 1990 to 2019. Between 1990 and 2019, SCI at neck level was more common than SCI below neck level in terms of incidence (492 thousand [354 to 675] *vs* 417 thousand [290 to 585]), prevalence (10·8 million [9·5 to 13·9] *vs* 9·7 million [9·2 to 10·4]), and YLDs (4·2 million [3·0 to 5·8] *vs* 1·9 million [1·3 to 2·5]). Falls (477 thousand [327 to 683] cases) and road injuries (230 thousand [122 to 389] cases) were the two leading causes of SCI globally in 2019.

**Interpretation:**

Although age-standardised rates of incidence, prevalence, and YLDs for SCI changed only slightly, absolute counts increased substantially from 1990 to 2019. Geographical heterogeneity in demographic, spatial, and temporal patterns of SCI, at both the national and regional levels, should be considered by policy makers aiming to reduce the burden of SCI.

**Funding:**

Bill & Melinda Gates Foundation.

## Introduction

Spinal cord injuries (SCIs) are associated with potential long-term disability, decreased life expectancy,^1^ reduced quality of life, and a great financial burden to health-care systems and the individuals who are affected.^2^, ^3^, ^4^ In the USA, for instance, SCI is estimated to cost more than US$9·7 billion annually.^5^, ^6^ In many previous studies, epidemiological data for SCI have been relatively heterogeneous, reporting the annual incidence as from 1·2 to 5·8 cases per 100 000 population.^7^ The annual incidence of SCI in developing countries (based on the definition of developing countries by the International Monetary Fund) consists of an even wider range, from 0·2 to 13·0 cases per 100 000 population.^3^, ^8^ This heterogeneity has been mainly rooted in diverse data-gathering methods, case-defining approaches, and socioeconomic structures of different countries.^9^, ^10^

Accurate and up-to-date estimates of the incidence and prevalence of disorders constitute the backbone of evidence-based health-care planning and resource allocation. Because no effective curative treatment for individuals with SCI has been identified,^11^, ^12^ prevention is crucial, and investigating epidemiological patterns would be the first step towards reaching this goal.^13^ To provide organised and targeted health-care support for people with SCI, it is also essential to have a comprehensive understanding of the prevalence and incidence of these injuries. Global data and comparisons between countries might help to improve understanding of the complexities and trends of SCI, and identifying patterns might help to show overlooked clustered environmental factors that can supplement and aid ongoing research.

**Research in context**

**Evidence before this study**

We searched PubMed, MEDLINE (In-Process and Other Non-Indexed Citations), and Embase for previous literature on spinal cord injuries (SCIs) published from database inception to March 17, 2023 (search strategies are available in the appendix p 12). We did not restrict the language of publications. Previous studies have mostly focused on traumatic SCI or the burden of injuries in a few locations, and data from low-income and middle-income countries are generally scarce. The Global Burden of Diseases, Injuries, and Risk Factors Study (GBD) collaborators previously published a paper on global, regional, and national burden of traumatic brain injury and SCI, according to the results from GBD 2016. GBD 2019 has made results for SCI available within the GBD online tool. We identified one paper published in 2022 that used GBD 2019 data to report on SCI, but this study did not include estimates broken down by sex or at the national level.

**Added value of this study**

Ours study expands our knowledge of the burden of SCI, particularly for locations not assessed previously. We retrieved estimates from GBD 2019, the largest systematic, data-driven, and most recent peer-reviewed assessment of the epidemiological burden of diseases by age group, sex, location, and cause of injury. GBD 2019 estimates replace those from previous GBD cycles, as in each iteration GBD generates revised estimates for the whole time series using the most up-to-date data and modelling methods. Compared with the paper published in 2022 that used GBD 2019 data, we tried to provide a comprehensive display of global, regional, and national results as well as trends by sex and age. To our knowledge, this is the first study from GBD collaborators to explore SCI exclusively, providing a comprehensive description of the incidence, prevalence, and years of life lived with disability and their trends over time stratified by age, sex, and cause of injury for 204 countries and territories over the period 1990–2019.

**Implications of the available evidence**

Our findings have the potential to facilitate health-care planning, especially in terms of guiding evidence-based prevention and resource allocation for the treatment of SCI. This study allows policy makers to prioritise locations and age groups that contribute most to the burdens of SCI. Furthermore, it provides crucial information for policy makers and medical professionals on epidemiological patterns of SCI. Our findings could help decision makers both on a national and on a global scale to promote practical preventive strategies against SCI.

The Global Burden of Diseases, Injuries, and Risk Factors Study (GBD) 2019 has developed systematic methods for quantifying health loss in detail by disease, age, sex, year, and geographical location.^14^ Previously, GBD collaborators published a paper on global, regional, and national burden of traumatic brain injury and spinal cord injury, according to the results from GBD 2016.^15^ Because GBD 2019 incorporates major data additions, improvements, and methodological refinements, our study updates and replaces previous GBD estimates. We aimed to quantify incidence, prevalence, and YLDs for SCI on the basis of sex, age group, level of injury, and cause, globally, regionally, and nationally, from 1990 to 2019. This manuscript was produced as part of the GBD Collaborator Network and in accordance with the GBD Protocol.

## Methods

### Overview

The detailed resources search, data processing steps, and modelling methods of GBD 2019 have been delineated in previous GBD studies.^14^, ^16^ Each step performed in this study to report data from the GBD database complied with the Guidelines for Accurate and Transparent Health Estimates Reporting (GATHER) statements.^17^ Our proposal for writing this manuscript was accepted by the GBD Scientific Publications team with the unique identifier 1925-GBD2019–112021. The University of Washington institutional review board committee approved GBD 2019, and the need for informed consent was waived because of the use of deidentified data.

### Definition of key terms and measurements

A detailed description of injury estimation has been published separately.^18^ In addition to prevalence and incidence, GBD uses YLDs to compare the morbidity associated with various non-fatal conditions, which is estimated by multiplying the prevalence counts with the disability weights for a given disease or injury.^16^ Disability weights range from 0, which is equivalent to perfect health, to 1, which is equivalent to death, and reflect the severity of that disease relative to all other health states. The term rate was used to indicate the number of cases per 100 000 population, in line with the other GBD reports.

### Estimation and modelling

Data for SCI were available for 93 (46%) of 204 countries for which GBD makes estimates. However, GBD makes predictions for all 204 locations through statistical tools that include predictive covariates and a geographical cascade, from which locations without data can borrow strength from surrounding locations. A detailed description of the GBD methods was published in the appendix of the GBD 2019 capstone paper on risk factors.^14^ The analytical cascade of DisMod-MR 2.1 software, a Bayesian meta-regression tool developed by the GBD team to run the estimations, is also shown in appendix 1 of the GBD 2017 capstone paper on incidence, prevalence, and YLDs.^19^

We used the SCI International Classification of Diseases (ICD) code list provided by the GBD modelling team for data processing. This list includes all conditions considered as SCI, both traumatic and non-traumatic, that were included in this study (appendix pp 2–6). For SCI, three categories are of interest in the GBD category hierarchy: spinal cord lesion at neck level, spinal cord lesion below neck level, and the parent category, spinal injuries. Our approach for estimating the burden of SCI was developed within the GBD 2019 methodology framework.^16^ We extracted detailed estimations for incidence, prevalence, and YLDs for SCI from the GBD Results Tool,^20^ at neck level, below neck level, and the aggregate of these injury causes with disaggregation of age, sex, year, and cause of injury. Estimates were retrieved for the world, 21 GBD regions, and 204 countries and territories. A detailed description of region and country categorisation is available in the appendix (p 10). We present estimates by number (ie, counts) and age-standardised rates per 100 000 population using the GBD standard population structure. We then compared the age-standardised rates between 1990 and 2019 and investigated temporal and spatial patterns according to age, sex, year, and geographical location. Global data for the cause of injury in 2019 are provided, in addition to changes from 1990. Annualised rates of change from 1990 to 2019 represent the mean percentage change per year during this period.

### Statistical analysis

Consistent with the GBD framework, we provide 95% uncertainty intervals (UIs) for all estimates, using the mean estimate across 1000 draws, with the 25th and 975th ranked values across all 1000 draws as the lower and upper bounds of the 95% UIs. The analysis contained in this Article is the result of an amalgamation of numerous surveys done with varying levels of quality and representativeness; we therefore report estimated results to a level of precision that conveys the true uncertainty of the GBD process. We present the count data in thousands and rates to one decimal place. We also used round outwards (ie, lower limit down and upper limit up) to maintain 95% uncertainty.

### Role of the funding source

The funder of the study had no role in study design, data collection, data analysis, data interpretation, or the writing of the report.

## Results

### Global overview

**Figure 1:**
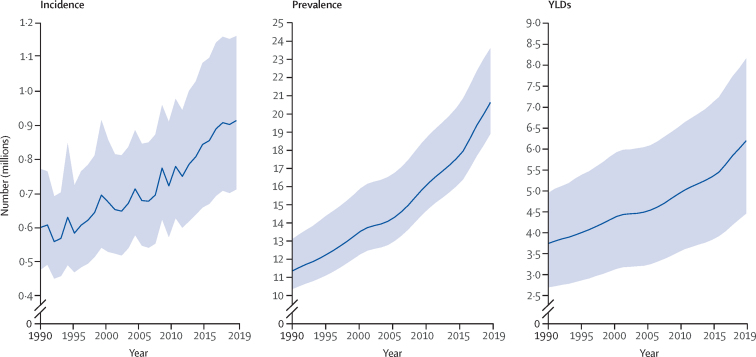
Global numbers of incidence, prevalence, and YLDs for spinal cord injuries, 1990–2019 Shading indicates 95% UIs. YLDs=years of life lived with disability.

**Figure 2:**
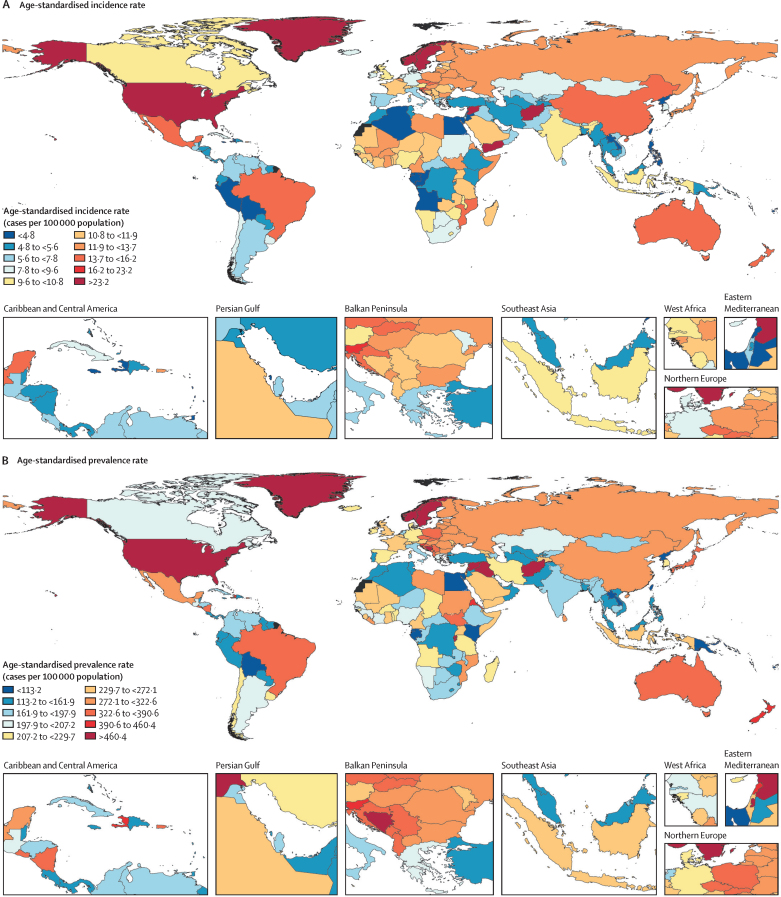

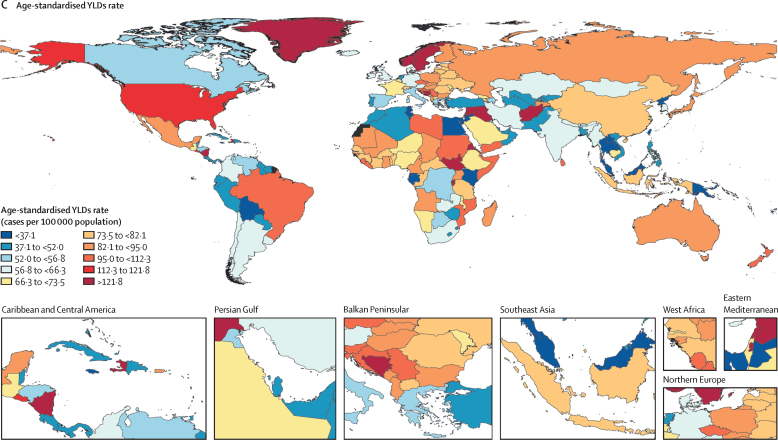
Global age-standardised incidence, prevalence, and YLDs rates per 100 000 population for spinal cord injuries, 2019 (A) Age-standardised incidence rate. (B) Age-standardised prevalence rate. (C) Age-standardised YLDs rate. Countries for which no data (and therefore no modelled results) are available are shown in black. YLDs=years of life lived with disability.

Globally, there were 20·6 million (95% UI 18·9 to 23·6) individuals with SCI in 2019 with an incidence of 0·9 million (95% UI 0·7 to 1·2) new cases (table). The YLDs attributable to SCI were estimated to be about 6·2 million (4·5 to 8·2) years. Globally, from 1990 to 2019, there was an 81·5% (74·2 to 87·1) increase in the prevalence of SCI, a 52·7% (30·3 to 69·8) increase in incidence, and a 65·4% (56·3 to 76·0) increase in YLDs (figure 1). Global age-standardised rates for SCI per 100 000 population in 2019 were 11·5 (8·9 to 14·6) for incidence, 253·0 (231·4 to 290·3) for prevalence, and 76·1 (54·7 to 100·3) for YLDs (figure 2). The global age-standardised rates per 100 000 population showed small changes in prevalence (5·8%, 2·6 to 9·5), incidence (–6·1%, –17·2 to 1·5), and YLDs (–1·5%, –5·5 to 3·2) from 1990 to 2019 (table).

**Table:** Incidence, prevalence, and YLDs for spinal cord injuries for all ages in 2019 and change in age-standardised rate per 100 000 population from 1990 to 2019 for 204 countries and regions

			**Incidence**			**Prevalence**			**YLDs**		
			1990 (thousands)	2019 (thousands)	Change in age-standardised rate (per 100 000), %	1990 (thousands)	2019 (thousands)	Change in age-standardised rate (per 100 000), %	1990 (thousands)	2019 (thousands)	Change in age-standardised rate (per 100 000), %
Global			595 (471 to 767)	909 (707 to 1156)	−6·1% (−17·2 to 1·5)	11 367 (10 375 to 13 112)	20 635 (18 926 to 23 611)	5·8% (2·6 to 9·5)	3749 (2703 to 4952)	6201 (4465 to 8156)	−1·5% (−5·5 to 3·2)
Central Asia, eastern Europe, and central Asia											
	Central Asia		5 (4 to 6)	6 (5 to 7)	−6·8% (−9·5 to −4·3)	115 (107 to 125)	175 (160 to 201)	−2·7% (−9·7 to 10·6)	39 (28 to 50)	55 (39 to 73)	−8·0% (−16·6 to 8·0)
		Armenia	0 (0 to 0)	0 (0 to 0)	−28·7% (−34·4 to −24·0)	16 (11 to 25)	9 (7 to 13)	−45·3% (−53·3 to −34·8)	5 (3 to 8)	3 (1·8 to 3·8)	−51·5% (−60·0 to −40·5)
		Azerbaijan	0 (0 to 0)	1 (0 to 1)	−10·7% (−17·4 to −5·2)	10 (9 to 11)	19 (17 to 24)	8·0% (−1·4 to 30·5)	4 (2 to 4)	6 (4 to 9)	0·6% (−14·2 to 25·7)
		Georgia	0 (0 to 0)	0 (0 to 0)	9·8% (4·9 to 15·0)	12 (11 to 12)	10 (9 to 12)	18·5% (8·9 to 40·7)	3 (2 to 5)	3 (2 to 4)	18·4% (2·9 to 47·0)
		Kazakhstan	1 (1 to 2)	1 (1 to 2)	−0·3% (−4·1 to 3·5)	31 (29 to 33)	39 (36 to 42)	1·0% (−1·4 to 3·1)	10 (7 to 13)	11 (8 to 15)	−9·5% (−17·4 to −1·4)
		Kyrgyzstan	0 (0 to 0)	0 (0 to 0)	−20·6% (−24·7 to −16·4)	7 (6 to 72)	10 (9 to 11)	−7·6% (−10·9 to −2·5)	2 (2 to 3)	3 (2 to 4)	−14·4% (−22·6 to −5·2)
		Mongolia	0 (0 to 0)	0 (0 to 0)	10·8% (3·6 to 16·7)	2 (2 to 3)	6 (6 to 7)	22·2% (18·4 to 26·0)	1 (1 to 1)	0 (0 to 0)	8·5% (−1·1 to 18·7)
		Tajikistan	0 (0 to 0)	0 (0 to 1)	−23·0% (−27·7 to −19·0)	7 (6 to 7)	21 (14 to 39)	43·7% (−1·3 to 165·1)	2 (2 to 3)	8 (4 to 16)	51·2% (−5·1 to 195·3)
		Turkmenistan	0 (0 to 0)	0 (0 to 0)	−2·3% (−7·9 to 3·1)	4 (4 to 4)	8 (7 to 8)	5·7% (0·9 to 13·4)	1 (1 to 2)	2 (18 to 3)	−2·9% (−13·2 to 10·5)
		Uzbekistan	1 (1 to 2)	2 (2 to 3)	3·0% (−1·0 to 6·9)	26 (24 to 28)	52 (49 to 56)	0·5% (−2·7 to 3·7)	9 (6 to 11)	16 (11 to 21)	−6·8% (−15·4 to 2·2)
	Central Europe		19 (15 to 23)	17 (14 to 21)	−12·9% (−16·1 to −9·8)	440 (413 to 472)	489 (456 to 534)	1·7% (−1·3 to 6·8)	134 (96 to 170)	132 (94 to 172)	−8·3% (−13·0 to −2·0)
		Albania	0 (0 to 0)	0 (0 to 0)	0·5% (−7·5 to 8·9)	9 (8 to 9)	11 (10 to 12)	8·6% (2·4 to 20·1)	3 (2 to 4)	3 (2 to 4)	−6·1% (−15·9 to 8·5)
		Bosnia and Herzegovina	1 (0 to 1)	0 (0 to 0)	−24·7% (−52·8 to −2·0)	14 (13 to 17)	20 (15 to 35)	62·4% (25·6 to 136·1)	49 (3 to 6)	6 (4 to 12)	54·5% (11·6 to 130·8)
		Bulgaria	1 (1 to 1)	1 (0 to 1)	−8·9% (−13·4 to −5·1)	33 (30 to 35)	27 (25 to 29)	−5·3% (−8·7 to −2·6)	99 (7 to 13)	7 (5 to 9)	−12·5% (−18·9 to −6·2)
		Croatia	1 (0 to 1)	1 (0 to 1)	0·3% (−7·1 to 8·0)	20 (18 to 21)	21 (20 to 23)	5·6% (0·1 to 14·3)	5 (4 to 7)	57 (4 to 7)	3·3% (−5·3 to 16·4)
		Czechia	2 (2 to 3)	2 (1 to 2)	−22·2% (−26·1 to −18·2)	42 (40 to 46)	51 (48 to 55)	1·0% (−2·5 to 5·0)	12 (9 to 16)	13 (9 to 17)	−5·6% (−11·8 to 1·3)
		Hungary	2 (2 to 3)	2 (1 to 2)	−28·0% (−31·8 to −24·0)	40 (39 to 43)	40 (37 to 44)	−4·9% (−8·6 to −0·1)	120 (8 to 15)	10 (7 to 14)	−13·8% (−20·6 to −6·4)
		Montenegro	0 (0 to 0)	0 (0 to 0)	0·5% (−2·2 to 3·3)	2 (2 to 2)	2 (2 to 3)	3·5% (0·3 to 6·9)	1 (0 to 1)	1 (0 to 1)	−2·5% (−8·5 to 4·3)
		North Macedonia	0 (0 to 0)	0 (0 to 0)	13·5% (6·2 to 21·2)	5 (5 to 6)	8 (7 to 8)	12·2% (8·3 to 16·6)	2 (1 to 2)	2 (1 to 3)	−1·1% (−9·3 to 7·5)
		Poland	6 (4 to 7)	6 (4 to 8)	−8·2% (−12·4 to −4·2)	132 (124 to 142)	164 (153 to 178)	1·4% (−1·1 to 4·0)	41 (29 to 52)	44 (31 to 56)	−11·0% (−15·5 to −6·7)
		Romania	3 (3 to 4)	2 (2 to 3)	−15·6% (−19·4 to −11·4)	83 (78 to 90)	73 (68 to 78)	−9·2% (−12·4 to −6·1)	26 (19 to 34)	20 (14 to 26)	−21·1% (−27·4 to −14·6)
		Serbia	1 (1 to 1)	1 (1 to 1)	0·6% (−6·2 to 7·5)	30 (28 to 32)	36 (32 to 45)	19·0% (5·6 to 49·8)	9 (7 to 12)	10 (7 to 14)	9·3% (−8·4 to 47·0)
		Slovakia	1 (0 to 1)	0 (0 to 1)	−7·4% (−11·8 to −3·5)	19 (17 to 20)	24 (23 to 26)	4·8% (1·8 to 8·4)	5 (4 to 7)	6 (4 to 8)	−4·7% (−11·3 to 2·0)
		Slovenia	0 (0 to 0)	0 (0 to 1)	−14·2% (−19·9 to −9·0)	9 (9 to 10)	11 (10 to 12)	−2·8% (−6·5 to 0·8)	2 (2 to 3)	3 (2 to 4)	−6·7% (−12·9 to −0·4)
	Eastern Europe		37 (29 to 46)	30 (24 to 38)	−15·6% (−18·3 to −12·9)	899 (838 to 972)	805 (746 to 876)	−13·8% (−16·4 to −9·0)	272 (193 to 347)	221 (156 to 283)	−21·3% (−25·1 to −15·3)
		Belarus	1 (1 to 2)	1 (1 to 2)	−3·6% (−10·5 to 3·2)	36 (33 to 38)	35 (33 to 38)	−6·0% (−8·7 to −3·2)	11 (8 to 14)	9 (6 to 12)	−17·1% (−23·2 to −11·2)
		Estonia	0 (0 to 0)	0 (0 to 0)	−34·4% (−39·2 to −30·1)	6 (6 to 7)	5 (4 to 5)	−23·9% (−26·9 to −20·8)	2 (1 to 2)	1 (0 to 2)	−31·0% (−36·6 to −25·5)
		Latvia	0 (0 to 1)	0 (0 to 0)	−36·5% (−40·1 to −32·9)	12 (11 to 13)	7 (6 to 7)	−28·5% (−31·2 to −25·9)	3 (2 to 4)	2 (1 to 2)	−35·1% (−40·2 to −29·7)
		Lithuania	0 (0 to 1)	0 (0 to 0)	−21·9% (−27·2 to −17·0)	15 (14 to 16)	11 (10 to 12)	−17·7% (−20·3 to −14·8)	4 (3 to 5)	3 (2 to 4)	−23·4% (−29·1 to −17·6)
		Moldova	0 (0 to 1)	0 (0 to 0)	−23·9% (−28·1 to −19·4)	13 (12 to 14)	11 (10 to 12)	−14·4% (−17·6 to −11·1)	4 (3 to 5)	3 (2 to 4)	−21·0% (−27·6 to −14·2)
		Russia	25 (19 to 32)	21 (17 to 27)	−16·2% (−19·6 to −12·6)	608 (566 to 658)	575 (532 to 628)	−13·4% (−16·4 to −8·1)	185 (131 to 235)	157 (112 to 203)	−21·5% (−25·8 to −14·9)
		Ukraine	8 (7 to 10)	6 (5 to 8)	−13·3% (−16·3 to −9·3)	209 (195 to 226)	161 (149 to 177)	−15·6% (−18·7 to −10·6)	62 (44 to 79)	44 (31 to 57)	−20·9% (−27·3 to −13·2)
High income											
	Australasia		3 (2 to 3)	5 (4 to 6)	8·1% (3·3 to 13·2)	74 (68 to 82)	128 (119 to 143)	6·8% (3·9 to 9·8)	20 (14 to 26)	34 (24 to 44)	6·6% (0·7 to 13·0)
		Australia	2 (2 to 3)	4 (3 to 5)	9·4% (4·0 to 15·2)	60 (55 to 67)	106 (98 to 118)	7·0% (3·9 to 10·4)	16 (11 to 21)	28 (19 to 36)	6·9% (−0·1 to 14·6)
		New Zealand	0 (0 to 0)	0 (0 to 1)	3·8% (−0·7 to 8·4)	14 (13 to 15)	22 (21 to 24)	7·0% (3·1 to 11·4)	4 (2 to 5)	6 (4 to 8)	6·5% (0·0 to 13·4)
	High-income Asia Pacific		24 (19 to 30)	33 (25 to 43)	−9·7% (−13·9 to −6·4)	624 (583 to 673)	859 (799 to 928)	−3·9% (−5·7 to −1·8)	171 (121 to 220)	227 (161 to 292)	−5·3% (−7·9 to −2·6)
		Brunei	0 (0 to 0)	0 (0 to 0)	−8·5% (−13·7 to −3·8)	0 (0 to 0)	0 (0 to 0)	−3·9% (−7·1 to −0·8)	0 (0 to 0)	0 (0 to 0)	−12·6% (−19·6 to −5·0)
		Japan	20 (15 to 25)	26 (20 to 35)	−7·7% (−11·8 to −4·2)	527 (490 to 569)	676 (622 to 733)	−1·4% (−3·4 to 0·8)	141 (100 to 183)	178 (125 to 230)	−0·8% (−3·5 to 1·9)
		Singapore	0 (0 to 0)	0 (0 to 0)	−7·0% (−10·5 to −3·4)	6 (5 to 6)	14 (13 to 15)	3·5% (0·7 to 6·5)	2 (1 to 2)	4 (3 to 5)	0·3% (−8·1 to 9·1)
		South Korea	4 (3 to 5)	6 (5 to 7)	−3·3% (−10·6 to 3·2)	91 (85 to 97)	168 (159 to 181)	7·9% (4·2 to 11·9)	28 (20 to 36)	45 (31 to 59)	−4·4% (−12·2 to 4·0)
	High-income North America		67 (53 to 87)	114 (85 to 156)	4·5% (−0·9 to 10·5)	1471 (1363 to 1596)	2142 (1966 to 2333)	−6·4% (−9·1 to −3·7)	383 (273 to 493)	543 (383 to 697)	−7·6% (−10·4 to −4·8)
		Canada	3 (2 to 3)	5 (4 to 7)	6·2% (0·6 to 11·9)	61 (58 to 66)	105 (98 to 112)	1·5% (−1·3 to 4·2)	16 (12 to 21)	27 (19 to 35)	0·8% (−7·1 to 9·0)
		Greenland	0 (0 to 0)	0 (0 to 0)	−16·9% (−20·5 to −13·6)	0 (0 to 0)	0 (0 to 0)	−11·1% (−13·8 to −8·7)	0 (0 to 0)	0 (0 to 0)	−17·6% (−21·9 to −12·8)
		USA	65 (50 to 84)	108 (81 to 149)	5·0% (−0·6 to 11·4)	1409 (1303 to 1532)	2037 (1865 to 2222)	−6·4% (−9·2 to −3·7)	367 (261 to 474)	516 (365 to 662)	−7·7% (−10·6 to −4·8)
	Southern Latin America		4 (3 to 4)	5 (4 to 6)	1·7% (−2·5 to 5·1)	93 (86 to 104)	153 (144 to 165)	7·7% (0·4 to 12·2)	30 (22 to 39)	44 (31 to 57)	−4·8% (−14·0 to 2·9)
		Argentina	2 (2 to 2)	3 (2 to 4)	−1·6% (−7·2 to 2·7)	63 (57 to 74)	99 (92 to 107)	3·8% (−5·3 to 9·4)	21 (15 to 28)	29 (20 to 38)	−7·2% (−19·1 to 3·5)
		Chile	0 (0 to 1)	1 (1 to 2)	9·3% (4·7 to 13·5)	22 (21 to 24)	45 (42 to 48)	18·1% (14·4 to 22·3)	7 (5 to 9)	12 (9 to 16)	1·3% (−7·4 to 10·4)
		Uruguay	0 (0 to 0)	0 (0 to 0)	0·0% (−6·7 to 6·4)	7 (6 to 7)	9 (8 to 9)	5·5% (1·9 to 9·0)	2 (1 to 3)	2 (2 to 3)	−3·1% (−11·2 to 6·3)
	Western Europe		46 (36 to 59)	61 (46 to 81)	−5·6% (−9·8 to −2·1)	1047 (982 to 1125)	1374 (1272 to 1486)	1·4% (−1·4 to 4·3)	280 (198 to 361)	362 (259 to 467)	1·3% (−2·2 to 5·1)
		Andorra	0 (0 to 0)	0 (0 to 0)	2·9% (−0·8 to 7·0)	0 (0 to 0)	0 (0 to 0)	6·9% (3·0 to 11·4)	0 (0 to 0)	0 (0 to 0)	6·5% (−2·0 to 15·3)
		Austria	1 (0 to 1)	1 (0 to 1)	−11·0% (−15·3 to −6·5)	22 (21 to 24)	28 (26 to 31)	−3·0% (−7·5 to 1·1)	6 (4 to 8)	7 (5 to 9)	−2·6% (−10·4 to 6·2)
		Belgium	1 (0 to 1)	2 (1 to 3)	13·7% (6·7 to 19·7)	29 (27 to 31)	42 (38 to 45)	11·4% (6·8 to 16·8)	7 (5 to 10)	11 (7 to 14)	10·8% (1·9 to 20·4)
		Cyprus	0 (0 to 0)	0 (0 to 0)	5·3% (−0·8 to 11·0)	1 (1 to 2)	3 (3 to 4)	10·5% (4·9 to 16·8)	0 (0 to 0)	0 (0 to 1)	3·9% (−4·9 to 13·5)
		Denmark	0 (0 to 0)	0 (0 to 0)	−23·4% (−27·8 to −18·7)	13 (13 to 15)	16 (14 to 17)	−0·6% (−5·4 to 4·5)	3 (2 to 5)	4 (3 to 5)	0·0% (−9·1 to 10·3)
		Finland	0 (0 to 1)	0 (0 to 1)	−8·9% (−13·3 to −4·7)	18 (17 to 19)	23 (21 to 26)	2·5% (−1·3 to 8·1)	5 (3 to 6)	6 (4 to 8)	3·1% (−4·9 to 12·3)
		France	8 (6 to 10)	11 (8 to 14)	−6·5% (−11·2 to −2·2)	164 (154 to 176)	222 (206 to 240)	1·3% (−2·4 to 5·3)	44 (32 to 57)	58 (41 to 76)	0·8% (−7·0 to 9·7)
		Germany	9 (7 to 11)	11 (8 to 15)	−1·3% (−6·3 to 3·3)	200 (187 to 215)	252 (231 to 274)	4·4% (−0·2 to 9·4)	53 (38 to 70)	66 (47 to 86)	4·3% (−4·6 to 13·4)
		Greece	0 (0 to 1)	0 (0 to 1)	−11·0% (−14·9 to −7·3)	26 (24 to 28)	28 (26 to 30)	−5·0% (−9·1 to −1·4)	7 (5 to 9)	7 (5 to 9)	−4·9% (−13·1 to 3·3)
		Iceland	0 (0 to 0)	0 (0 to 0)	−1·9% (−6·7 to 2·1)	0 (0 to 0)	0 (0 to 1)	3·8% (−0·2 to 8·4)	0 (0 to 0)	0 (0 to 0)	4·0% (−4·7 to 13·7)
		Ireland	0 (0 to 0)	0 (0 to 0)	−0·8% (−6·2 to 3·9)	7 (7 to 8)	13 (12 to 14)	9·4% (5·0 to 13·8)	2 (1 to 2)	3 (2 to 4)	8·3% (−0·6 to 17·7)
		Israel	0 (0 to 0)	0 (0 to 0)	−10·8% (−28·3 to −0·1)	9 (8 to 10)	23 (19 to 32)	29·7% (11·9 to 69·2)	2 (2 to 3)	7 (4 to 10)	33·1% (8·1 to 83·4)
		Italy	7 (5 to 9)	7 (5 to 9)	−23·5% (−26·1 to −20·8)	152 (143 to 165)	167 (156 to 181)	−11·7% (−14·2 to −8·8)	40 (29 to 52)	44 (32 to 57)	−10·6% (−13·9 to −6·8)
		Luxembourg	0 (0 to 0)	0 (0 to 0)	−7·5% (−13·0 to −2·4)	1 (1 to 1)	2 (1 to 2)	−1·8% (−6·1 to 2·3)	0 (0 to 0)	0 (0 to 0)	−2·4% (−10·8 to 5·8)
		Malta	0 (0 to 0)	0 (0 to 0)	6·2% (2·9 to 9·7)	0 (0 to 0)	1 (1 to 1)	15·3% (10·8 to 20·4)	0 (0 to 0)	0 (0 to 0)	12·7% (3·7 to 23·2)
		Monaco	0 (0 to 0)	0 (0 to 0)	12·1% (7·6 to 16·1)	0 (0 to 0)	0 (0 to 0)	13·9% (9·9 to 19·0)	0 (0 to 0)	0 (0 to 0)	13·8% (6·7 to 21·3)
		Netherlands	1 (1 to 2)	2 (2 to 3)	21·0% (12·7 to 29·3)	30 (28 to 32)	45 (42 to 49)	10·4% (6·0 to 14·5)	8 (6 to 10)	12 (8 to 15)	10·0% (0·5 to 19·8)
		Norway	1 (1 to 2)	2 (1 to 3)	−1·2% (−5·4 to 2·7)	25 (23 to 27)	36 (32 to 39)	3·5% (0·5 to 6·9)	7 (4 to 8)	9 (6 to 12)	3·8% (0·2 to 7·8)
		Portugal	0 (0 to 1)	0 (0 to 1)	−21·9% (−27·9 to −16·8)	23 (21 to 27)	23 (22 to 26)	−24·7% (−30·4 to −20·7)	7 (5 to 9)	6 (4 to 8)	−31·4% (−39·3 to −24·0)
		San Marino	0 (0 to 0)	0 (0 to 0)	9·2% (3·5 to 14·3)	0 (0 to 0)	0 (0 to 0)	11·2% (7·1 to 15·7)	0 (0 to 0)	0 (0 to 0)	10·7% (3·9 to 18·0)
		Spain	3 (2 to 4)	4 (3 to 6)	−0·9% (−9·9 to 7·6)	87 (82 to 93)	129 (120 to 140)	5·6% (−0·3 to 11·6)	23 (16 to 30)	34 (24 to 45)	6·0% (−4·4 to 17·5)
		Sweden	2 (2 to 4)	4 (2 to 6)	1·8% (−2·9 to 6·5)	53 (48 to 58)	72 (65 to 79)	5·2% (2·0 to 8·9)	14 (10 to 18)	19 (13 to 25)	5·6% (−0·1 to 11·3)
		Switzerland	1 (0 to 1)	1 (1 to 2)	−18·4% (−21·9 to −14·8)	25 (23 to 27)	32 (30 to 35)	−9·2% (−13·2 to −4·0)	6 (4 to 8)	8 (6 to 11)	−8·1% (−15·9 to 0·0)
		UK	6 (5 to 8)	9 (7 to 12)	5·9% (0·4 to 10·5)	155 (144 to 167)	210 (195 to 228)	5·0% (2·2 to 8·0)	41 (29 to 53)	55 (39 to 71)	3·9% (0·6 to 7·1)
		England	4 (3 to 5)	6 (4 to 8)	4·6% (−0·8 to 9·2)	109 (102 to 117)	145 (134 to 158)	3·0% (−0·1 to 6·9)	29 (21 to 37)	38 (27 to 49)	2·3% (−1·0 to 5·9)
		Northern Ireland	0 (0 to 0)	0 (0 to 0)	4·5% (−16·5 to 16·8)	7 (6 to 8)	12 (10 to 13)	15·2% (9·3 to 21·8)	2 (1 to 2)	3 (2 to 4)	14·1% (5·2 to 23·5)
		Scotland	1 (0 to 1)	1 (1 to 2)	9·0% (3·1 to 14·5)	24 (22 to 27)	33 (30 to 37)	9·5% (4·7 to 15·0)	7 (5 to 9)	8 (6 to 11)	6·3% (−1·0 to 14·2)
		Wales	0 (0 to 0)	0 (0 to 1)	16·7% (10·3 to 22·6)	14 (12 to 15)	20 (18 to 28)	14·1% (9·5 to 19·0)	3 (2 to 5)	5 (3 to 7)	13·7% (6·3 to 21·5)
Latin America and Caribbean											
	Andean Latin America		3 (2 to 5)	3 (2 to 3)	−39·5% (−66·5 to −11·1)	45 (36 to 68)	84 (74 to 105)	−1·2% (−13·1 to 7·9)	17 (11 to 29)	27 (18 to 38)	−17·2% (−29·4 to −6·1)
		Bolivia	0 (0 to 0)	0 (0 to 0)	2·5% (−3·2 to 7·8)	5 (4 to 5)	11 (10 to 12)	10·4% (6·7 to 14·1)	2 (1 to 2)	4 (3 to 5)	−4·1% (−14·4 to 7·2)
		Ecuador	0 (0 to 0)	0 (0 to 1)	14·6% (9·9 to 19·3)	10 (9 to 12)	23 (22 to 25)	5·3% (−1·9 to 11·2)	4 (3 to 5)	7 (5 to 9)	−10·4% (−20·5 to 0·7)
		Peru	2 (1 to 5)	1 (1 to 2)	−56·2% (−78·8 to −23·6)	30 (21 to 52)	50 (41 to 70)	−5·3% (−19·6 to 7·4)	12 (7 to 23)	16 (10 to 25)	−21·7% (−35·2 to −6·6)
	Caribbean		2 (2 to 3)	3 (3 to 4)	8·1% (0·9 to 15·1)	45 (43 to 48)	118 (93 to 165)	64·7% (29·1 to 131·2)	15 (11 to 19)	38 (25 to 57)	66·0% (24·3 to 140·0)
		Antigua and Barbuda	0 (0 to 0)	0 (0 to 0)	11·6% (6·4 to 17·2)	0 (0 to 0)	0 (0 to 0)	16·1% (13·1 to 19·5)	0 (0 to 0)	0 (0 to 0)	7·0% (−3·6 to 18·9)
		The Bahamas	0 (0 to 0)	0 (0 to 0)	8·0% (0·8 to 14·5)	0 (0 to 0)	0 (0 to 0)	14·7% (10·6 to 18·7)	0 (0 to 0)	0 (0 to 0)	7·2% (−4·3 to 19·7)
		Barbados	0 (0 to 0)	0 (0 to 0)	9·5% (3·6 to 15·6)	0 (0 to 0)	0 (0 to 0)	12·0% (8·3 to 15·3)	0 (0 to 0)	0 (0 to 0)	2·6% (−8·5 to 14·3)
		Belize	0 (0 to 0)	0 (0 to 0)	−9·4% (−41·0 to 13·7)	0 (0 to 0)	0 (0 to 0)	16·5% (12·3 to 21·4)	0 (0 to 0)	0 (0 to 0)	8·6% (−1·1 to 19·7)
		Bermuda	0 (0 to 0)	0 (0 to 0)	9·3% (−1·1 to 20·4)	0 (0 to 0)	0 (0 to 0)	19·7% (15·2 to 24·9)	0 (0 to 0)	0 (0 to 0)	6·9% (−1·7 to 16·5)
		Cuba	0 (0 to 0)	1 (0 to 2)	17·7% (6·3 to 28·5)	16 (15 to 17)	23 (22 to 25)	8·2% (3·6 to 12·3)	5 (3 to 6)	6 (5 to 8)	−2·0% (−10·8 to 8·3)
		Dominica	0 (0 to 0)	0 (0 to 0)	7·8% (3·3 to 12·2)	0 (0 to 0)	0 (0 to 0)	22·5% (9·8 to 39·4)	0 (0 to 0)	0 (0 to 0)	15·0% (−0·4 to 32·9)
		Dominican Republic	0 (0 to 0)	0 (0 to 0)	31·7% (26·3 to 37·6)	6 (6 to 6)	15 (14 to 15)	28·4% (24·4 to 32·8)	2 (2 to 3)	5 (3 to 6)	15·6% (4·1 to 28·6)
		Grenada	0 (0 to 0)	0 (0 to 0)	21·7% (14·1 to 28·6)	0 (0 to 0)	0 (0 to 0)	−2·0% (−29·8 to 20·0)	0 (0 to 0)	0 (0 to 0)	−13·6% (−39·9 to 12·2)
		Guyana	0 (0 to 0)	0 (0 to 0)	14·9% (11·5 to 18·3)	0 (0 to 0)	0 (0 to 1)	18·4% (14·7 to 21·7)	0 (0 to 0)	0 (0 to 0)	10·3% (−1·1 to 22·8)
		Haiti	0 (0 to 0)	0 (0 to 0)	−0·2% (−5·4 to 3·7)	4 (4 to 5)	50 (26 to 93)	390·9% (164·9 to 805·3)	2 (1 to 2)	18 (9 to 34)	354·6% (143·5 to 723·3)
		Jamaica	0 (0 to 0)	0 (0 to 0)	−5·1% (−22·3 to 5·0)	2 (2 to 2)	3 (3 to 4)	1·0% (−1·7 to 3·8)	0 (0 to 1)	1 (0 to 1)	−5·1% (−16·2 to 5·6)
		Puerto Rico	0 (0 to 0)	0 (0 to 0)	5·9% (−3·0 to 15·7)	11 (10 to 11)	15 (14 to 17)	12·5% (5·7 to 25·1)	3 (2 to 4)	4 (3 to 5)	1·6% (−7·8 to 14·7)
		Saint Kitts and Nevis	0 (0 to 0)	0 (0 to 0)	6·8% (0·1 to 13·2)	0 (0 to 0)	0 (0 to 0)	21·0% (17·0 to 25·0)	0 (0 to 0)	0 (0 to 0)	9·5% (−1·5 to 21·2)
		Saint Lucia	0 (0 to 0)	0 (0 to 0)	0·8% (−13·6 to 10·3)	0 (0 to 0)	0 (0 to 0)	17·6% (13·2 to 21·8)	0 (0 to 0)	0 (0 to 0)	6·4% (−4·4 to 18·8)
		Saint Vincent and the Grenadines	0 (0 to 0)	0 (0 to 0)	13·6% (8·1 to 19·1)	0 (0 to 0)	0 (0 to 0)	14·9% (10·3 to 19·8)	0 (0 to 0)	0 (0 to 0)	8·8% (−2·7 to 22·3)
		Suriname	0 (0 to 0)	0 (0 to 0)	13·3% (7·7 to 18·5)	0 (0 to 0)	0 (0 to 0)	−1·0% (−23·1 to 10·6)	0 (0 to 0)	0 (0 to 0)	−8·6% (−31·4 to 7·6)
		Trinidad and Tobago	0 (0 to 0)	0 (0 to 0)	−19·8% (−45·0 to −1·2)	3 (1 to 1)	2 (2 to 2)	17·3% (13·5 to 20·3)	0 (0 to 0)	0 (0 to 0)	8·0% (−3·9 to 21·0)
		Virgin Islands	0 (0 to 0)	0 (0 to 0)	4·3% (−3·6 to 12·2)	0 (0 to 0)	0 (0 to 0)	−3·4% (−6·9 to 0·1)	0 (0 to 0)	0 (0 to 0)	−12·4% (−19·5 to −4·6)
	Central Latin America		20 (15 to 24)	25 (20 to 31)	−23·4% (−34·2 to −17·9)	422 (355 to 564)	656 (594 to 762)	−16·7% (−24·4 to −11·9)	153 (105 to 230)	200 (143 to 263)	−28·1% (−36·4 to −22·5)
		Colombia	3 (2 to 4)	3 (2 to 3)	−40·6% (−58·4 to −27·3)	62 (54 to 74)	100 (86 to 132)	−10·5% (−18·7 to 3·4)	22 (15 to 29)	30 (20 to 44)	−20·9% (−31·5 to −5·7)
		Costa Rica	0 (0 to 0)	0 (0 to 0)	−0·5% (−4·1 to 3·7)	4 (3 to 4)	8 (8 to 9)	7·5% (4·5 to 10·5)	1 (0 to 1)	2 (2 to 3)	−1·7% (−9·6 to 6·6)
		El Salvador	0 (0 to 0)	0 (0 to 0)	−61·9% (−83·5 to −24·9)	34 (12 to 91)	20 (12 to 42)	−49·7% (−59·1 to −25·9)	14 (5 to 41)	7 (3 to 16)	−58·1% (−65·6 to −38·0)
		Guatemala	0 (0 to 0)	1 (0 to 1)	−44·2% (−72·0 to −10·7)	22 (10 to 49)	30 (23 to 47)	−35·5% (−53·5 to −4·6)	9 (4 to 23)	10 (7 to 18)	−46·5% (−61·1 to −18·9)
		Honduras	0 (0 to 0)	0 (0 to 0)	7·1% (1·0 to 14·2)	4 (4 to 5)	14 (12 to 17)	32·3% (18·9 to 56·1)	2 (1 to 2)	5 (3 to 6)	18·5% (2·2 to 42·6)
		Mexico	12 (9 to 16)	17 (13 to 23)	−16·1% (−17·6 to −14·6)	241 (223 to 265)	405 (374 to 442)	−12·6% (−14·4 to −11·0)	83 (60 to 105)	120 (86 to 154)	−23·4% (−26·2 to −20·8)
		Nicaragua	0 (0 to 0)	0 (0 to 0)	−13·9% (−33·3 to −1·3)	25 (8 to 67)	20 (10 to 44)	−48·7% (−56·3 to −26·6)	11 (3 to 31)	7 (3 to 18)	−56·2% (−62·5 to −38·8)
		Panama	0 (0 to 0)	0 (0 to 0)	−5·8% (−10·3 to −0·8)	3 (3 to 5)	6 (6 to 8)	−4·2% (−21·6 to 4·6)	1 (0 to 2)	2 (1 to 3)	−15·0% (−33·7 to −0·7)
		Venezuela	1 (0 to 1)	2 (1 to 2)	−6·7% (−13·0 to −0·4)	26 (24 to 28)	53 (48 to 58)	9·1% (2·4 to 19·3)	9 (6 to 11)	16 (11 to 20)	−4·5% (−14·9 to 8·0)
	Tropical Latin America		21 (16 to 28)	33 (25 to 44)	−8·1% (−11·5 to −5·0)	433 (398 to 474)	796 (734 to 869)	−2·5% (−5·4 to 0·3)	147 (105 to 186)	237 (170 to 302)	−12·8% (−16·6 to −9·0)
		Brazil	21 (16 to 27)	33 (25 to 44)	−8·1% (−11·5 to −5·0)	429 (394 to 470)	787 (725 to 859)	−2·4% (−5·3 to 0·4)	145 (103 to 184)	234 (168 to 299)	−12·7% (−16·5 to −8·8)
		Paraguay	0 (0 to 0)	0 (0 to 0)	6·0% (2·3 to 9·7)	4 (4 to 5)	9 (8 to 9)	0·2% (−3·4 to 3·3)	1 (1 to 2)	3 (2 to 4)	−9·6% (−19·0 to 0·3)
North Africa and Middle East			43 (28 to 65)	53 (35 to 98)	−32·6% (−60·7 to 8·9)	72 (412 to 1682)	1 (1 to 3)	5·0% (−19·4 to 21·3)	304 (139 to 722)	564 (290 to 1229)	−5·4% (−27·6 to 12·3)
	Afghanistan		1 (0 to 3)	10 (3 to 30)	308·3% (115·4 to 502·2)	170 (21 to 675)	229 (65 to 655)	−47·9% (−65·8 to 41·5)	77 (9 to 311)	99 (26 to 287)	−50·7% (−67·4 to 32·9)
	Algeria		1 (0 to 1)	2 (1 to 2)	−6·7% (−13·1 to −0·4)	25 (23 to 28)	68 (56 to 95)	20·0% (2·4 to 61·7)	9 (6 to 11)	21 (14 to 33)	12·2% (−11·6 to 62·6)
	Bahrain		0 (0 to 0)	0 (0 to 0)	5·5% (−2·2 to 13·3)	0 (0 to 0)	2 (2 to 3)	16·1% (8·9 to 22·2)	0 (0 to 0)	0 (0 to 0)	−2·1% (−13·7 to 11·7)
	Egypt		2 (1 to 2)	4 (3 to 5)	16·2% (6·5 to 33·7)	40 (38 to 43)	92 (85 to 100)	13·8% (8·4 to 21·8)	14 (10 to 18)	29 (20 to 37)	0·6% (−11·7 to 16·8)
	Iran		25 (13 to 45)	4 (3 to 5)	−90·2% (−94·7 to −81·2)	153 (88 to 316)	192 (147 to 290)	−37·1% (−55·4 to −15·0)	58 (29 to 128)	59 (37 to 97)	−48·2% (−62·4 to −29·6)
	Iraq		2 (1 to 3)	3 (2 to 5)	−32·3% (−44·7 to −15·7)	141 (45 to 400)	275 (100 to 735)	−23·2% (−32·6 to −11·6)	60 (18 to 170)	106 (35 to 295)	−28·5% (−37·1 to −18·3)
	Jordan		0 (0 to 0)	0 (0 to 0)	−1·0% (−6·6 to 4·5)	3 (2 to 3)	12 (11 to 14)	10·8% (5·7 to 19·4)	0 (0 to 1)	3 (2 to 5)	−3·1% (−14·6 to 11·4)
	Kuwait		0 (0 to 0)	0 (0 to 0)	−86·2% (−94·8 to −60·5)	2 (2 to 3)	9 (7 to 12)	12·5% (3·7 to 28·4)	0 (0 to 1)	2 (1 to 4)	4·4% (−10·1 to 20·9)
	Lebanon		0 (0 to 0)	0 (0 to 0)	−71·5% (−87·7 to −37·5)	25 (6 to 81)	21 (8 to 61)	−50·2% (−57·6 to −27·9)	10 (2 to 33)	7 (2 to 20)	−58·0% (−63·8 to −41·4)
	Libya		0 (0 to 0)	0 (0 to 1)	141·5% (40·4 to 405·7)	5 (4 to 9)	21 (13 to 41)	71·0% (31·1 to 126·1)	2 (1 to 4)	7 (3 to 16)	67·9% (21·5 to 123·8)
	Morocco		1 (0 to 1)	2 (1 to 2)	7·9% (2·8 to 13·6)	27 (24 to 33)	49 (45 to 53)	4·0% (−6·5 to 10·4)	10 (7 to 14)	16 (11 to 20)	−9·1% (−21·3 to 2·6)
	Oman		0 (0 to 0)	0 (0 to 0)	−0·5% (−7·3 to 6·9)	2 (2 to 2)	7 (6 to 8)	9·2% (5·4 to 13·6)	0 (0 to 0)	2 (1 to 3)	−7·7% (−17·9 to 4·0)
	Palestine		0 (0 to 0)	0 (0 to 0)	−66·8% (−83·5 to −29·6)	13 (4 to 34)	35 (12 to 93)	16·0% (1·3 to 23·9)	5 (1 to 15)	13 (4 to 37)	9·3% (−5·8 to 17·8)
	Qatar		0 (0 to 0)	0 (0 to 0)	−3·1% (−10·3 to 3·4)	0 (0 to 0)	5 (5 to 6)	−0·5% (−4·4 to 3·4)	0 (0 to 0)	1 (1 to 2)	−17·5% (−26·7 to −8·2)
	Saudi Arabia		1 (0 to 1)	4 (3 to 5)	33·2% (26·1 to 40·3)	24 (22 to 25)	99 (90 to 109)	27·2% (21·8 to 32·5)	8 (5 to 10)	28 (19 to 37)	6·1% (−3·8 to 16·6)
	Sudan		3 (1 to 6)	3 (2 to 4)	−43·5% (−72·0 to −7·9)	32 (26 to 44)	89 (69 to 136)	31·4% (19·6 to 50·5)	12 (8 to 19)	32 (20 to 55)	22·1% (5·5 to 42·5)
	Syria		0 (0 to 0)	3 (1 to 7)	548·2% (131·0 to 1510·3)	16 (9 to 39)	147 (46 to 408)	567·8% (215·4 to 1184·7)	6 (2 to 15)	57 (16 to 160)	579·1% (218·2 to 1263·5)
	Tunisia		0 (0 to 0)	0 (0 to 0)	6·1% (−0·9 to 12·9)	8 (7 to 9)	17 (15 to 180)	8·5% (3·7 to 13·4)	3 (1 to 3)	5 (3 to 6)	−5·6% (−16·0 to 5·5)
	Türkiye		3 (2 to 4)	4 (3 to 5)	11·1% (−8·9 to 28·0)	52 (49 to 56)	139 (121 to 172)	47·8% (32·9 to 75·6)	18 (13 to 23)	40 (27 to 57)	24·1% (3·7 to 55·4)
	United Arab Emirates		0 (0 to 0)	0 (0 to 0)	−3·4% (−8·2 to 1·3)	2 (2 to 3)	17 (16 to 18)	−1·2% (−5·0 to 1·7)	0 (0 to 1)	5 (3 to 7)	−11·0% (−21·2 to −1·0)
	Yemen		0 (0 to 0)	9 (3 to 24)	510·4% (122·0 to 1565·0)	16 (10 to 30)	68 (34 to 160)	74·0% (24·9 to 134·0)	6 (3 to 13)	27 (12 to 66)	70·7% (18·8 to 131·0)
South Asia			81 (64 to 103)	156 (120 to 201)	5·0% (−7·7 to 12·7)	1313 (1235 to 1396)	3161 (2953 to 3413)	22·8% (19·4 to 27·0)	495 (364 to 612)	1076 (780 to 1355)	11·8% (7·6 to 16·9)
	Bangladesh		5 (3 to 6)	8 (6 to 11)	4·3% (−3·6 to 11·0)	85 (77 to 94)	229 (204 to 266)	41·0% (33·7 to 50·7)	33 (23 to 42)	77 (55 to 100)	24·0% (10·9 to 38·1)
	Bhutan		0 (0 to 0)	0 (0 to 0)	28·3% (20·1 to 38·7)	0 (0 to 0)	1 (1 to 1)	43·5% (37·8 to 50·8)	0 (0 to 0)	0 (0 to 0)	22·8% (10·6 to 37·1)
	India		71 (56 to 91)	134 (104 to 174)	2·0% (−11·3 to 10·4)	1134 (1065 to 1209)	2621 (2458 to 2801)	18·5% (15·6 to 21·4)	426 (313 to 527)	887 (644 to 1113)	7·9% (4·1 to 11·5)
	Nepal		1 (0 to 1)	2 (1 to 2)	10·9% (7·1 to 14·3)	18 (16 to 19)	51 (44 to 63)	50·5% (35·4 to 80·4)	7 (5 to 9)	18 (12 to 25)	37·5% (17·1 to 70·7)
	Pakistan		4 (3 to 6)	11 (8 to 15)	21·6% (14·8 to 28·2)	76 (72 to 81)	259 (229 to 318)	51·5% (37·8 to 77·6)	29 (21 to 36)	93 (66 to 127)	42·7% (25·1 to 72·6)
Southeast Asia, east Asia, and Oceania											
	East Asia		104 (79 to 134)	236 (173 to 315)	39·8% (32·0 to 48·0)	2175 (2017 to 2348)	5148 (4799 to 5551)	37·9% (34·0 to 42·3)	742 (531 to 941)	1389 (980 to 1794)	11·1% (4·9 to 16·8)
		China	103 (78 to 132)	234 (172 to 313)	40·8% (32·9 to 49·1)	2140 (1983 to 2312)	5096 (4750 to 5499)	38·6% (34·7 to 43·1)	731 (523 to 926)	1374 (968 to 1775)	11·4% (5·1 to 17·2)
		North Korea	0 (0 to 0)	0 (0 to 1)	−9·3% (−16·0 to −1·5)	14 (13 to 15)	22 (21 to 23)	−3·4% (−7·1 to 0·7)	5 (3 to 6)	7 (5 to 9)	−5·2% (−16·1 to 6·8)
		Taiwan (province of China)	0 (0 to 1)	1 (0 to 1)	−23·5% (−27·2 to −19·7)	21 (19 to 22)	30 (28 to 32)	−14·2% (−18·3 to −10·0)	6 (4 to 8)	8 (58 to 10)	−19·9% (−28·7 to −10·2)
	Oceania		0 (0 to 0)	0 (0 to 0)	10·2% (−2·6 to 18·9)	4 (3 to 4)	11 (10 to 13)	25·9% (19·5 to 36·0)	1 (1 to 2)	4 (3 to 5)	21·8% (9·5 to 35·9)
		American Samoa	0 (0 to 0)	0 (0 to 0)	6·3% (0·0 to 13·0)	0 (0 to 0)	0 (0 to 0)	16·1% (8·1 to 29·6)	0 (0 to 0)	0 (0 to 0)	9·7% (−1·7 to 25·4)
		Cook Islands	0 (0 to 0)	0 (0 to 0)	−11·6% (−27·8 to 3·0)	0 (0 to 0)	0 (0 to 0)	11·4% (6·7 to 16·3)	0 (0 to 0)	0 (0 to 0)	2·4% (−8·2 to 14·1)
		Federated States of Micronesia	0 (0 to 0)	0 (0 to 0)	11·7% (6·2 to 17·5)	0 (0 to 0)	0 (0 to 0)	19·5% (14·0 to 28·3)	0 (0 to 0)	0 (0 to 0)	9·8% (−3·6 to 24·9)
		Fiji	0 (0 to 0)	0 (0 to 0)	7·3% (2·6 to 11·6)	0 (0 to 0)	0 (0 to 0)	13·3% (9·8 to 17·5)	0 (0 to 0)	0 (0 to 0)	9·0% (−2·8 to 23·2)
		Guam	0 (0 to 0)	0 (0 to 0)	2·3% (−4·2 to 9·0)	0 (0 to 0)	0 (0 to 0)	4·9% (0·5 to 9·0)	0 (0 to 0)	0 (0 to 0)	1·8% (−6·7 to 10·3)
		Kiribati	0 (0 to 0)	0 (0 to 0)	2·7% (−1·9 to 7·4)	0 (0 to 0)	0 (0 to 0)	16·3% (10·5 to 25·9)	0 (0 to 0)	0 (0 to 0)	13·3% (−3·8 to 34·3)
		Marshall Islands	0 (0 to 0)	0 (0 to 0)	6·6% (2·7 to 10·9)	0 (0 to 0)	0 (0 to 0)	7·0% (4·5 to 9·6)	0 (0 to 0)	0 (0 to 0)	2·3% (−8·9 to 13·9)
		Nauru	0 (0 to 0)	0 (0 to 0)	15·1% (8·3 to 22·5)	0 (0 to 0)	0 (0 to 0)	14·7% (10·7 to 19·2)	0 (0 to 0)	0 (0 to 0)	9·7% (−1·2 to 21·3)
		Niue	0 (0 to 0)	0 (0 to 0)	19·6% (10·9 to 28·6)	0 (0 to 0)	0 (0 to 0)	24·8% (19·2 to 32·6)	0 (0 to 0)	0 (0 to 0)	17·4% (4·4 to 32·0)
		Northern Mariana Islands	0 (0 to 0)	0 (0 to 0)	6·9% (−3·6 to 17·5)	0 (0 to 0)	0 (0 to 0)	5·1% (0·7 to 9·5)	0 (0 to 0)	0 (0 to 0)	−1·4% (−9·5 to 7·1)
		Palau	0 (0 to 0)	0 (0 to 0)	17·1% (11·8 to 21·9)	0 (0 to 0)	0 (0 to 0)	17·0% (13·5 to 20·7)	0 (0 to 0)	0 (0 to 0)	9·0% (−2·3 to 21·3)
		Papua New Guinea	0 (0 to 0)	0 (0 to 0)	13·7% (−4·3 to 25·0)	2 (2 to 2)	8 (7 to 9)	35·9% (27·2 to 49·4)	0 (0 to 1)	3 (2 to 4)	29·8% (12·8 to 50·0)
		Samoa	0 (0 to 0)	0 (0 to 0)	−20·0% (−39·0 to −4·2)	0 (0 to 0)	0 (0 to 0)	52·2% (31·4 to 89·8)	0 (0 to 0)	0 (0 to 0)	44·1% (19·0 to 79·8)
		Solomon Islands	0 (0 to 0)	0 (0 to 0)	14·7% (8·6 to 20·4)	0 (0 to 0)	0 (0 to 0)	14·4% (8·0 to 19·4)	0 (0 to 0)	0 (0 to 0)	7·7% (−5·5 to 21·5)
		Tokelau	0 (0 to 0)	0 (0 to 0)	21·5% (13·2 to 30·4)	0 (0 to 0)	0 (0 to 0)	24·7% (20·4 to 29·6)	0 (0 to 0)	0 (0 to 0)	13·4% (1·9 to 25·3)
		Tonga	0 (0 to 0)	0 (0 to 0)	−8·5% (−18·6 to 0·0)	0 (0 to 0)	0 (0 to 0)	6·6% (2·3 to 11·6)	0 (0 to 0)	0 (0 to 0)	1·6% (−11·3 to 16·3)
		Tuvalu	0 (0 to 0)	0 (0 to 0)	18·7% (11·0 to 26·8)	0 (0 to 0)	0 (0 to 0)	23·7% (19·6 to 29·0)	0 (0 to 0)	0 (0 to 0)	13·4% (1·5 to 27·0)
		Vanuatu	0 (0 to 0)	0 (0 to 0)	10·2% (6·4 to 14·3)	0 (0 to 0)	0 (0 to 0)	19·5% (12·6 to 28·4)	0 (0 to 0)	0 (0 to 0)	15·2% (0·2 to 31·8)
	Southeast Asia		37 (28 to 48)	47 (38 to 60)	−18·6% (−31·8 to −10·7)	691 (603 to 899)	1249 (1128 to 1450)	1·2% (−10·6 to 9·3)	259 (180 to 372)	412 (298 to 546)	−9·2% (−22·3 to −0·6)
		Cambodia	0 (0 to 1)	0 (0 to 0)	−12·1% (−45·5 to 16·4)	30 (9 to 99)	27 (17 to 58)	−42·3% (−62·5 to 8·7)	13 (3 to 43)	10 (5 to 22)	−51·1% (−67·5 to −7·8)
		Indonesia	18 (14 to 24)	25 (19 to 33)	−11·4% (−13·6 to −9·4)	371 (342 to 415)	624 (577 to 681)	−3·3% (−9·0 to 1·7)	138 (102 to 173)	210 (153 to 266)	−11·5% (−17·8 to −5·9)
		Laos	0 (0 to 0)	0 (0 to 0)	−44·7% (−72·4 to −14·2)	3 (2 to 3)	6 (5 to 6)	11·3% (8·2 to 14·3)	1 (0 to 1)	2 (1 to 3)	−0·3% (−11·3 to 13·0)
		Malaysia	0 (0 to 0)	2 (1 to 2)	11·3% (6·6 to 16·3)	15 (14 to 16)	38 (36 to 41)	13·0% (10·3 to 15·7)	5 (4 to 7)	12 (8 to 15)	−1·0% (−11·3 to 9·7)
		Maldives	0 (0 to 0)	0 (0 to 0)	9·1% (0·5 to 17·5)	0 (0 to 0)	0 (0 to 0)	39·1% (29·9 to 55·0)	0 (0 to 0)	0 (0 to 0)	12·0% (−3·0 to 28·5)
		Mauritius	0 (0 to 0)	0 (0 to 0)	22·5% (13·7 to 32·3)	2 (2 to 2)	4 (3 to 4)	27·6% (22·5 to 33·6)	0 (0 to 0)	1 (0 to 1)	16·5% (6·4 to 28·0)
		Myanmar	2 (1 to 3)	3 (2 to 3)	−3·8% (−10·5 to 3·1)	46 (31 to 99)	95 (72 to 135)	40·0% (−11·8 to 104·0)	19 (10 to 43)	33 (22 to 50)	21·5% (−27·3 to 83·5)
		Philippines	4 (3 to 6)	5 (4 to 6)	−38·2% (−54·1 to −22·5)	70 (54 to 111)	127 (109 to 165)	−8·8% (−24·0 to 1·4)	27 (17 to 47)	44 (30 to 63)	−16·0% (−31·3 to −5·3)
		Seychelles	0 (0 to 0)	0 (0 to 0)	9·6% (5·2 to 14·1)	0 (0 to 0)	0 (0 to 0)	16·5% (12·3 to 20·3)	0 (0 to 0)	0 (0 to 0)	4·2% (−5·0 to 13·6)
		Sri Lanka	4 (1 to 11)	1 (1 to 2)	−72·0% (−86·6 to −38·6)	29 (20 to 48)	72 (44 to 143)	72·2% (36·6 to 118·9)	11 (6 to 21)	24 (12 to 52)	58·3% (18·6 to 104·7)
		Thailand	3 (2 to 4)	4 (3 to 5)	−10·4% (−17·0 to −3·8)	68 (63 to 73)	112 (105 to 122)	−4·5% (−8·1 to 0·0)	23 (17 to 29)	32 (23 to 41)	−18·5% (−27·2 to −8·6)
		Timor–Leste	0 (0 to 0)	0 (0 to 0)	−77·1% (−91·0 to −42·1)	3 (1 to 7)	4 (1542 to 9215)	−4·6% (−10·6 to 3·9)	1 (0 to 3)	1 (0 to 4)	−13·8% (−21·0 to −3·4)
		Viet Nam	3 (2 to 3)	6 (4 to 7)	26·2% (19·3 to 33·7)	53 (50 to 57)	136 (128 to 144)	28·9% (25·6 to 32·7)	19 (14 to 24)	41 (30 to 53)	10·6% (−0·8 to 22·4)
Sub-Saharan Africa											
	Central sub-Saharan Africa		3 (2 to 7)	5 (4 to 8)	−29·1% (−47·9 to −10·3)	55 (34 to 110)	158 (93 to 330)	30·3% (16·9 to 44·5)	24 (13 to 51)	65 (34 to 145)	25·2% (8·7 to 40·0)
		Angola	2 (0 to 5)	0 (0 to 1)	−77·0% (−90·8 to −43·8)	32 (11 to 87)	46 (23 to 107)	−34·2% (−42·0 to −15·1)	15 (5 to 41)	19 (8 to 46)	−40·7% (−48·0 to 25·1)
		Central African Republic	0 (0 to 0)	0 (0 to 0)	93·0% (24·9 to 269·5)	1 (1 to 1)	9 (4 to 22)	224·5% (63·5 to 623·9)	0 (0 to 0)	4 (2 to 10)	263·5% (71·3 to 728·9)
		Congo(Brazzaville)	0 (0 to 0)	0 (0 to 0)	−6·1% (−8·9 to −3·3)	1 (1 to 1)	9 (4 to 20)	191·0% (54·9 to 531·0)	0 (0 to 0)	4 (1 to 9)	208·5% (54·6 to 598·2)
		Democratic Republic of the Congo	1 (1 to 2)	4 (2 to 6)	17·8% (3·4 to 51·4)	19 (18 to 21)	92 (59 to 180)	90·4% (29·3 to 249·7)	8 (5 to 10)	38 (21 to 78)	99·1% (25·3 to 273·4)
		Equatorial Guinea	0 (0 to 0)	0 (0 to 0)	2·7% (−2·8 to 7·9)	0 (0 to 0)	0 (0 to 0)	26·1% (22·1 to 30·3)	0 (0 to 0)	0 (0 to 0)	7·6% (−5·1 to 22·0)
		Gabon	0 (0 to 0)	0 (0 to 0)	−1·2% (−4·5 to 2·8)	0 (0 to 0)	1 (1 to 1)	6·4% (4·1 to 8·9)	0 (0 to 0)	0 (0 to 0)	−0·6% (−11·6 to 11·4)
	Eastern sub-Saharan Africa		55 (23 to 134)	29 (22 to 38)	−69·7% (−86·8 to −36·7)	323 (219 to 604)	713 (537 to 1156)	8·3% (−3·2 to 15·3)	137 (79 to 292)	280 (176 to 503)	1·0% (−10·3 to 8·8)
		Burundi	0 (0 to 0)	0 (0 to 0)	−0·8% (−6·9 to 10·1)	3 (3 to 3)	67 (22 to 189)	799·7% (206·9 to 2316·4)	1 (0 to 2)	30 (9 to 85)	883·8% (217·8 to 2582·5)
		Comoros	0 (0 to 0)	0 (0 to 0)	2·6% (−1·1 to 6·8)	0 (0 to 0)	2 (1 to 2)	15·7% (12·9 to 18·5)	0 (0 to 0)	0 (0 to 0)	7·1% (−0·2 to 14·5)
		Djibouti	0 (0 to 0)	0 (0 to 0)	−21·0% (−47·9 to −2·2)	0 (0 to 0)	3 (2 to 3)	10·3% (5·6 to 20·0)	0 (0 to 0)	0 (0 to 0)	3·2% (−6·3 to 15·5)
		Eritrea	3 (0 to 10)	0 (0 to 0)	−89·2% (−96·4 to −59·6)	27 (8 to 77)	23 (13 to 51)	−53·5% (−64·7 to −19·5)	13 (3 to 39)	9 (4 to 23)	−58·7% (−68·2 to −30·1)
		Ethiopia	38 (11 to 107)	4 (3 to 5)	−93·3% (−97·5 to −77·9)	76 (41 to 165)	141 (87 to 285)	6·0% (−7·0 to 29·3)	34 (16 to 81)	57 (30 to 122)	−4·1% (−17·5 to 20·0)
		Kenya	0 (0 to 1)	2 (1 to 2)	0·3% (−1·4 to 2·5)	14 (13 to 15)	41 (37 to 49)	12·9% (5·7 to 29·9)	5 (4 to 6)	15 (11 to 20)	11·1% (2·0 to 31·2)
		Madagascar	1 (0 to 2)	2 (2 to 3)	−2·7% (−7·0 to 2·4)	18 (17 to 20)	45 (41 to 49)	3·2% (0·3 to 6·1)	7 (5 to 9)	17 (12 to 22)	−0·5% (−8·3 to 8·6)
		Malawi	0 (0 to 1)	1 (1 to 2)	2·3% (−2·3 to 7·0)	11 (10 to 12)	26 (24 to 28)	17·0% (13·4 to 20·6)	4 (3 to 5)	10 (7 to 12)	11·8% (3·3 to 20·9)
		Mozambique	2 (1 to 3)	3 (2 to 5)	−7·9% (−43·0 to 18·4)	49 (25 to 116)	55 (47 to 69)	−39·6% (−64·2 to −6·3)	22 (10 to 57)	21 (15 to 29)	−47·9% (−69·7 to −14·4)
		Rwanda	1 (0 to 3)	1 (0 to 2)	−50·5% (−74·6 to −23·1)	12 (11 to 13)	62 (32 to 142)	157·8% (37·1 to 467·9)	5 (3 to 6)	25 (11 to 62)	159·4% (28·4 to 491·6)
		Somalia	1 (0 to 3)	2 (1 to 3)	−39·4% (−61·1 to −10·5)	12 (8 to 33)	39 (28 to 65)	19·0% (−34·3 to 71·1)	5 (3 to 14)	16 (10 to 31)	16·4% (−38·4 to 74·1)
		South Sudan	0 (0 to 0)	0 (0 to 1)	6·3% (−5·0 to 34·6)	12 (9 to 19)	24 (17 to 41)	23·2% (8·9 to 45·7)	5 (3 to 8)	10 (6 to 18)	22·8% (4·3 to 47·0)
		Tanzania	2 (1 to 3)	5 (4 to 6)	0·1% (−3·9 to 4·2)	34 (3 to 37)	90 (84 to 98)	16·6% (14·1 to 19·1)	13 (9 to 16)	33 (23 to 41)	11·5% (3·5 to 20·7)
		Uganda	2 (1 to 2)	3 (3 to 5)	−7·6% (−27·4 to 3·3)	43 (22 to 100)	69 (60 to 90)	−16·5% (−49·7 to 25·6)	18 (8 to 47)	26 (18 to 37)	−25·7% (−56·3 to 17·9)
		Zambia	0 (0 to 0)	1 (1 to 2)	3·8% (−2·0 to 9·4)	9 (8 to 9)	25 (23 to 27)	18·7% (14·8 to 22·5)	3 (2 to 4)	9 (6 to 11)	9·1% (0·3 to 18·6)
	Southern sub-Saharan Africa		6 (3 to 6)	6 (5 to 9)	−11·9% (−16·4 to −8·3)	88 (81 to 101)	125 (117 to 135)	−20·3% (−25·3 to −17·5)	32 (23 to 40)	42 (30 to 52)	−25·2% (−30·8 to −21·5)
		Botswana	0 (0 to 0)	0 (0 to 0)	12·0% (6·2 to 17·3)	1 (1 to 2)	4 (3 to 4)	13·3% (10·4 to 16·4)	0 (0 to 0)	1 (1 to 2)	1·5% (−7·6 to 10·9)
		Eswatini	0 (0 to 0)	0 (0 to 0)	13·6% (9·6 to 18·0)	0 (0 to 0)	1 (1 to 1)	−3·0% (−5·2 to −0·7)	0 (0 to 0)	0 (0 to 0)	−9·0% (−17·1 to −0·9)
		Lesotho	0 (0 to 0)	0 (0 to 0)	20·5% (12·5 to 29·0)	2 (2 to 2)	3 (2 to 3)	−4·6% (−7·5 to −1·2)	0 (0 to 0)	0 (0 to 0)	−8·8% (−18·1 to 1·3)
		Namibia	0 (0 to 0)	0 (0 to 0)	8·1% (4·6 to 11·7)	3 (2 to 6)	4 (3 to 5)	−22·6% (−48·3 to 1·5)	1 (0 to 2)	1 (1 to 2)	−34·3% (−58·3 to −9·7)
		South Africa	3 (2 to 5)	5 (3 to 6)	−18·8% (−23·8 to −15·2)	70 (64 to 80)	97 (90 to 104)	−24·0% (−29·4 to −21·0)	25 (18 to 32)	31 (22 to 40)	−29·4% (−35·3 to −25·5)
		Zimbabwe	1 (0 to 1)	1 (1 to 1)	12·6% (1·9 to 32·6)	11 (10 to 12)	17 (15 to 18)	−7·3% (−9·4 to −4·9)	4 (2 to 5)	6 (4 to 8)	−5·5% (−13·3 to 4·0)
	Western sub-Saharan Africa		16 (12 to 22)	38 (28 to 52)	3·3% (−5·4 to 7·9)	246 (228 to 266)	687 (626 to 784)	17·7% (14·0 to 26·0)	92 (68 to 115)	248 (181 to 325)	12·7% (7·8 to 22·6)
		Benin	0 (0 to 0)	1 (0 to 1)	5·8% (2·3 to 9·2)	6 (54 to 6)	17 (16 to 19)	11·0% (8·9 to 13·4)	2 (1 to 3)	6 (4 to 8)	6·8% (−1·8 to 15·2)
		Burkina Faso	0 (0 to 0)	2 (1 to 3)	28·1% (18·4 to 49·0)	10 (9 to 11)	32 (30 to 34)	32·0% (29·4 to 34·7)	4 (2 to 5)	12 (8 to 15)	27·4% (17·5 to 38·4)
		Cameroon	0 (0 to 0)	3 (2 to 4)	15·9% (9·2 to 27·1)	15 (14 to 17)	49 (44 to 54)	6·7% (2·3 to 12·4)	6 (4 to 7)	18 (13 to 23)	1·3% (−7·4 to 11·3)
		Cape Verde	0 (0 to 0)	0 (0 to 0)	24·8% (20·3 to 29·6)	0 (0 to 0)	1 (1 to 1)	22·3% (19·4 to 25·4)	0 (0 to 0)	0 (0 to 0)	9·2% (0·0 to 18·8)
		Chad	0 (0 to 0)	1 (0 to 2)	−11·6% (−39·3 to 10·5)	10 (7 to 18)	22 (19 to 30)	−2·6% (−23·5 to 14·9)	4 (2 to 9)	9 (6 to 13)	−8·4% (−28·8 to 13·2)
		Côte d'Ivoire	0 (0 to 0)	2 (1 to 3)	2·3% (−0·8 to 5·6)	15 (13 to 16)	39 (36 to 43)	12·6% (9·8 to 17·8)	5 (4 to 7)	15 (10 to 19)	10·7% (1·9 to 21·0)
		The Gambia	0 (0 to 0)	0 (0 to 0)	10·5% (7·3 to 13·6)	1 (1 to 1)	3 (3 to 3)	1·3% (−12·1 to 8·9)	0 (0 to 0)	1 (0 to 1)	−4·4% (−19·9 to 7·6)
		Ghana	1 (0 to 2)	3 (2 to 5)	19·5% (16·0 to 23·5)	21 (19 to 23)	63 (57 to 68)	23·2% (20·7 to 25·8)	8 (5 to 10)	22 (16 to 28)	16·1% (7·7 to 24·7)
		Guinea	0 (0 to 0)	1 (0 to 1)	14·7% (10·8 to 18·5)	8 (7 to 8)	18 (16 to 19)	15·8% (12·5 to 20·7)	3 (2 to 4)	7 (5 to 9)	11·3% (2·0 to 21·5)
		Guinea–Bissau	0 (0 to 0)	0 (0 to 0)	0·7% (−2·2 to 3·8)	1 (1 to 1)	3 (3 to 3)	14·4% (9·3 to 24·1)	0 (0 to 0)	1 (0 to 2)	9·5% (0·0 to 23·1)
		Liberia	1 (0 to 4)	0 (0 to 0)	−85·9% (−95·1 to −53·4)	3 (2 to 4)	10 (7 to 18)	41·5% (14·8 to 87·6)	1 (0 to 2)	4 (2 to 8)	37·0% (7·0 to 82·0)
		Mali	0 (0 to 1)	2 (1 to 3)	−1·2% (−11·5 to 5·5)	10 (9 to 11)	34 (28 to 48)	36·6% (18·1 to 81·6)	4 (3 to 5)	13 (9 to 20)	33·8% (10·0 to 88·8)
		Mauritania	0 (0 to 0)	0 (0 to 0)	−8·8% (−15·5 to −3·4)	3 (3 to 4)	8 (7 to 8)	6·4% (4·6 to 8·1)	1 (0 to 2)	2 (2 to 3)	−2·7% (−9·4 to 4·1)
		Niger	0 (0 to 0)	2 (1 to 2)	2·7% (−2·7 to 6·6)	9 (8 to 9)	27 (25 to 29)	12·7% (9·9 to 16·2)	3 (2 to 4)	10 (7 to 13)	6·6% (−1·6 to 16·0)
		Nigeria	7 (5 to 9)	17 (12 to 22)	9·2% (4·8 to 17·8)	113 (106 to 122)	307 (281 to 351)	16·7% (11·9 to 27·7)	41 (29 to 51)	107 (78 to 141)	11·4% (5·0 to 25·7)
		São Tomé and Principe	0 (0 to 0)	0 (0 to 0)	23·9% (19·7 to 28·1)	0 (0 to 0)	0 (0 to 0)	22·9% (19·2 to 26·0)	0 (0 to 0)	0 (0 to 0)	13·3% (4·8 to 21·6)
		Senegal	0 (0 to 0)	1 (0 to 2)	3·0% (−5·7 to 8·2)	9 (8 to 10)	23 (21 to 25)	16·3% (13·4 to 20·3)	3 (2 to 4)	8 (6 to 10)	10·9% (3·2 to 21·1)
		Sierra Leone	0 (0 to 0)	0 (0 to 0)	10·6% (7·2 to 14·0)	4 (4 to 5)	16 (13 to 24)	54·6% (24·6 to 126·6)	2 (1 to 2)	6 (4 to 10)	55·4% (19·0 to 138·7)
		Togo	0 (0 to 0)	0 (0 to 1)	4·6% (1·4 to 7·6)	5 (4 to 5)	13 (12 to 14)	6·1% (3·7 to 8·9)	2 (1 to 2)	5 (3 to 6)	2·5% (−4·9 to 10·6)

Where super-regions contain only one region, data for the region are listed in the header row for the super-region. 95% UI=95% uncertainty interval. YLDs=years of life lived with disability.

### Regional and national trends

Regionally, high-income North America had the highest age-standardised incidence rate in 2019 with 22 (95% UI 17 to 29) cases per 100 000 population, followed by 14 (11 to 17) per 100 000 in Australasia and 14 (10 to 19) per 100 000 in tropical Latin America (appendix p 7). Andean Latin America (4 [3 to 5] per 100 000) and central sub-Saharan Africa (4 [3 to 6] per 100 000) had the lowest age-standardised incidence rates. East Asia had the greatest increase in age-standardised incidence rate from 1990 to 2019, whereas the greatest decreases were shown in eastern sub-Saharan Africa and Andean Latin America (table). The highest age-standardised prevalence rates in 2019 were identified in high-income North America (437 [95% UI 404–474] per 100 000), Australasia (362 [331–405] per 100 000), and central Europe (332 [308–363] per 100 000; appendix p 7). By contrast, Oceania, with 103 (95–116) per 100 000, and Andean Latin America, with 135 (120–169) per 100 000, had the lowest prevalence rates. The Caribbean and east Asia had the greatest increases from 1990 to 2019 in age-standardised prevalence rates (table). Southern sub-Saharan Africa and central Latin America had the greatest decreases from 1990 to 2019 in age-standardised prevalence rates (table). The highest YLDs rates in 2019 were identified in high-income North America, with 113 (95% UI 80 to 146) YLDs per 100 000, followed by Australasia (96 [67–127] per 100 000) and tropical Latin America (96 [69–123] per 100 000; appendix p 7). The lowest age-standardised YLDs rates were identified for Oceania (36 [26–46] per 100 000) and Andean Latin America (43 [30–61] per 100 000). The Caribbean had the greatest increase in age-standardised YLDs rates and central Latin America had the greatest decrease in age-standardised YLDs rates from 1990 to 2019 (table; appendix p 7).

Incidence, prevalence, YLDs, and the corresponding percentage changes in age-standardised rates for SCI varied across countries between 1990 and 2019 (table). Age-standardised incidence rates of SCI in 2019 varied widely between countries, with the highest rates observed in Afghanistan (43·7 [95% UI 11·8–131·7] per 100 000) and Greenland (31·9 [24·1–42·6] per 100 000) versus the lowest rates in North Korea (3·0 [2·4–3·8] per 100 000), and Kiribati (3·0 [2·5–3·8] per 100 000; figure 2A). The greatest national increases in the age-standardised incidence rates per 100 000 population for SCI from 1990 to 2019 were for Syria, Yemen, Afghanistan, and Libya. By contrast, the greatest national decreases in the age-standardised incidence rates per 100 000 population from 1990 to 2019 were for Ethiopia, Iran, Eritrea, and Kuwait (table). The age-standardised prevalence rates of SCI in 2019 varied from 60·4 (95% UI 55·5–66·3) per 100 000 in Kiribati, and 69·9 (66·0–74·4) per 100 000 in North Korea to 813·8 (289·2–2164·1) per 100 000 in Palestine, and 1048·9 (332·6–2942·0) per 100 000 in Syria (figure 2B). Burundi, Syria, Haiti, Central African Republic, Congo (Brazzaville), and Rwanda had the greatest increases in the age-standardised prevalence rates of SCI from 1990 to 2019, whereas Eritrea, Lebanon, El Salvador, Nicaragua, and Afghanistan had the greatest decreases in the age-standardised prevalence rates (table). The highest age-standardised YLDs rates of SCI in 2019 were for Syria, with 403·1 (95% UI 112·5–1150·3) per 100 000, and Afghanistan, with 341·9 (87·3–1016·4) per 100 000. By contrast, the lowest age-standardised YLDs rates were for Kiribati, with 22·4 (16·1–28·9) per 100 000, and North Korea, with 22·5 (16·1–29·0) per 100 000 (figure 2C). Burundi, Syria, Haiti, Central African Republic, Congo (Brazzaville), and Rwanda had the greatest increases in the age-standardised YLDs rate from 1990 to 2019, respectively. By contrast, Eritrea, El Salvador, and Lebanon had the greatest decreases.

### Age-sex-specific patterns

**Figure 3:**
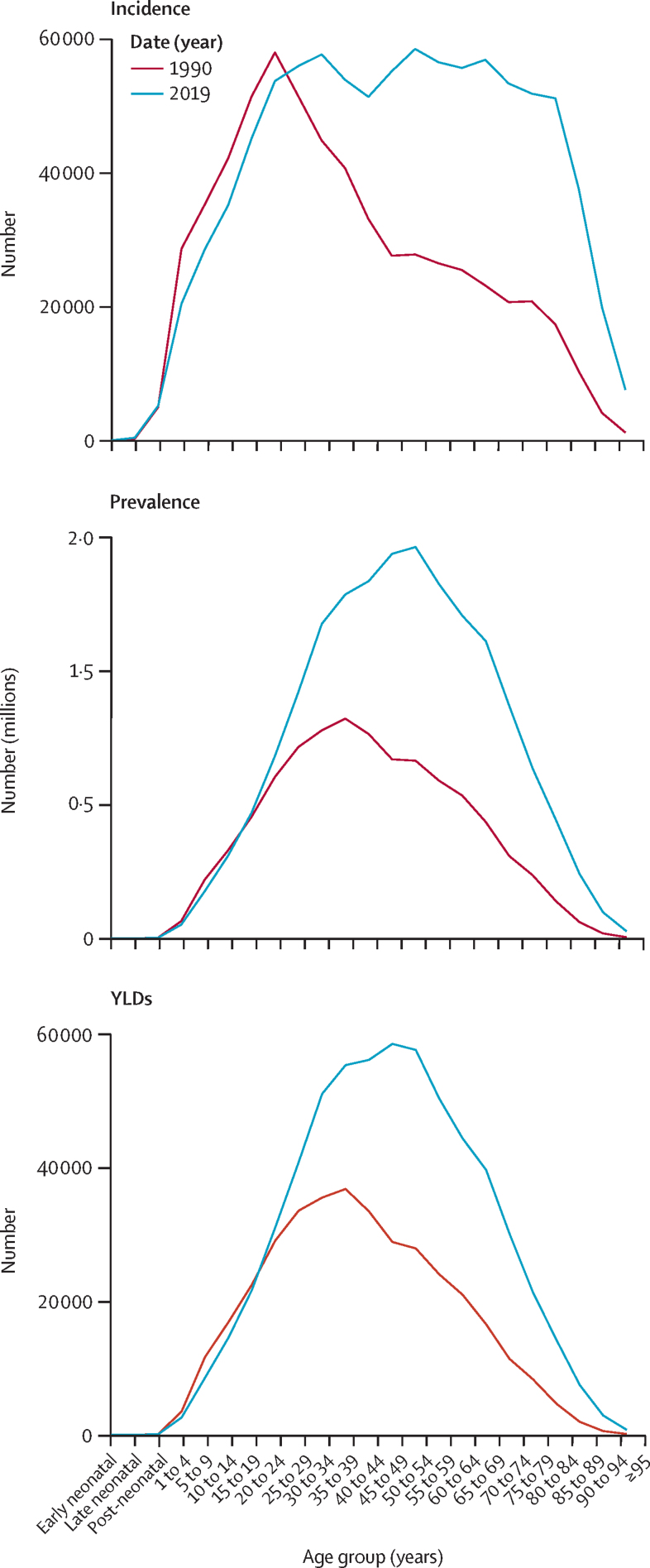
Global numbers of incidence, prevalence, and YLDs for spinal cord injuries from all causes according to age group, 1990–2019 Early neonatal corresponds to the first week after birth, late neonatal corresponds to 1–4 weeks after birth, and post-neonatal corresponds to 4 weeks to 12 months after birth.

Distribution of global incidence, prevalence, and YLDs for SCI from all causes in 2019 according to age groups is shown in the appendix (p 8). Compared with 1990, the incidence chart shows an important difference: one peak exists at age 20–24 years in 1990, whereas in 2019, incidence increases with age and remains higher than 50 000 cases from around age 20 years to around age 84 years, with two peaks, at age 30–34 years and age 50–54 years, after which it decreases (figure 3). The prevalence and YLDs charts show similar patterns and changes. In 1990, both prevalence and YLDs had one peak at the age of 35–39 years, whereas in 2019, the prevalence chart shows a higher peak at around 50–54 years and the YLD chart shows a higher peak at around 45–59 years (figure 3). Global incidence of SCI in children and people younger than 25 years seems to have been lower in 2019 than in 1990.

Global trends of incidence, prevalence, and YLD numbers for SCIs from 1990 to 2019 for males, females, and male and female sexes combined are shown in the appendix (pp 8, 10). Globally, there were 9·2 million (95% UI 8·4–10·7) cases of SCI in females and 11·5 million (10·5–13·0) cases in males in 2019, with age-standardised prevalence rates of 220·2 (95% UI 201·0–257·2) per 100 000 person-years for females and 284·3 (261·0–321·9) per 100 000 person-years for males. In 2019, there were 485 thousand (95% UI 386–609) new cases of SCI in males and 423 thousand (321–555) new cases in females (ie, 53·4% of the global incidence was in males). In 1990, there were 339 thousand (269–434) new cases in males and 256 thousand (201–336) new cases in females (ie, 57·0% of the global incidence was in males). The age-standardised incidence rates were 12·5 (95% UI 10·0–15·7) per 100 000 person-years for males and 10·2 (7·8–13·4) per 100 000 person-years for females. Global YLDs of SCI in 2019 were 2·7 million (95% UI 1·9–3·6) in females and 3·5 million (2·5–4·6) in males. Global age-standardised YLD rates per 100 000 population for SCI in 2019 were 64·6 (95% UI 46·4–87·3) for females and 87·3 (62·6–113·8) for males.

### Injury patterns

In 2019, the global incidence of spinal cord lesions at neck level was 492 thousand (95% UI 354–675) and the incidence of spinal cord lesions below neck level was 417 thousand (290–585) new cases (ie, 54·2% at neck level *vs* and 45·8% below neck level). Prevalence of spinal cord lesions at neck level was 10·8 million (95% UI 9·5–13·9) cases, compared with 9·7 million (9·2–10·4) cases below neck level (52·7% at neck level *vs* 47·3% below neck level). YLDs attributable to SCI differed between spinal cord lesions at neck level (4·2 million, 95% UI 3·0–5·8) and spinal cord lesions below neck level (1·9 million, 1·3–2·5; 68·5% at neck level *vs* 31·5% below level). The incidence, prevalence, and YLDs for SCI from 1990 to 2019 according to the level of injury (ie, spinal cord lesions at neck level *vs* spinal cord lesions below neck level) are shown in the appendix (p 11).

Globally, the two leading causes of SCI in 2019 were falls (477 thousand [95% UI 327–683] cases) and road injuries (including motor vehicle, motorcyclist, cyclist, pedestrian, and other road injuries; 230 thousand [122–389] cases; appendix p 9). The highest age-standardised incidence rates among the causes of SCI globally in 2019 were falls (6·2 [95% UI 4·2–8·8] cases per 100 000 population) and road injuries (3·0 [1·6–5·0] per 100 000). The global age-standardised rate change of incidence, prevalence, and YLDs for the two leading causes of SCI (falls and road injuries) from 1990 to 2019 are shown in the appendix (p 11). The global incidence, prevalence, and YLDs and age-standardised rate per 100 000 population for SCI in 2019, according to the cause of injury, are shown in the appendix (p 9).

## Discussion

From 1990 to 2019, the global numbers of incidence, prevalence, and YLDs for SCI increased substantially. However, their age-standardised rates showed only slight changes. The increases in prevalence and YLDs might be attributable to several factors, including population growth, ageing, and improvements in global health-care access and quality.^21^ According to the GBD 2019 injuries hierarchy, SCIs are the type of injury that causes the highest mean long-term disability (ie, measured by YLDs). In this categorisation, spinal cord lesion below neck level is ranked first in terms of level of disability, and spinal cord lesion at neck level is ranked fourth.^16^ Because a major proportion of prevalence and YLD estimates come from chronic disabling conditions, SCI could therefore contribute to a major proportion of the burden of disease among all injuries. Although life expectancy of individuals with SCI, even with optimal medical management, is lower than that of the general population, several long-term studies have suggested that mortality for people with SCI has decreased since 1944.^22^, ^23^ However, this effect might not be true for people from low-income and middle-income countries (LMICs) who cannot afford the long-term treatment and care.^21^ The increase of more than 80% in prevalence from 1990 to 2019 according to our study could be a reflection of increasing life expectancy and population ageing. However, the increase in absolute incidence, which is the main cause of the increasing YLDs and prevalence, warrants consideration. Whereas traumatic cases of SCI are more common in young people (eg, aged 15–29 years) and older people (eg, aged ≥65 years),^24^ the incidence of non-traumatic SCI steadily increases with age. According to our results, the peaks of incidence, prevalence, and YLDs are shifting towards older ages over time, and an ageing population with increased lifespan can consequently lead to the increase in the absolute number of SCIs.

Although data for Socio-demographic Index (SDI) is not currently available for SCI in the GBD database, owing to the absence of statistical support for presenting such data, our findings support previous research indicating that incidence, prevalence, and YLDs of SCI are higher in high-income regions than in lower-income regions. High-income North America and Australasia, for example, had the highest age-standardised incidence, prevalence, and YLD rates in 2019. One reason for this finding might be the high quality of data coming from these regions, in addition to the number of reports and methods of reporting. In a study by Tropeano and colleagues,^25^ among 2304 articles from 2008 to 2018, North America (843 [36·6%]) and Europe (833 [36·2%]) were the major publishing regions for studies of traumatic SCI. A systematic review in 2018 by Kumar and colleagues^26^ showed the incidence of traumatic SCI to be higher in LMICs (13·6 cases per 100 000 people) than in high-income countries (8·7 cases per 100 000 people). This wide variation might reflect true regional differences in the incidence of SCI, but also the quality of studies included in the analysis. A deeper look into the data at a national level could help to improve the understanding of these differences. The four countries with the greatest increase in age-standardised incidence rates from 1990 to 2019 (ie, Syria, Yemen, Afghanistan, and Libya) are located in North Africa and the Middle East and were all involved in war and conflicts during this period. As depicted in the GBD 2016 paper,^15^ North Africa and the Middle East was the only GBD region in which conflict and terrorism had a greater contribution than falls to age-standardised incidence of SCI. The wide UIs in these countries also reflect the heterogeneity of data sources for the estimates of SCI. We conceptualise that the burden of SCI, as assessed with counts of incidence, prevalence, and YLDs, is increasing worldwide, and it is essential to consider local epidemiological data in planning preventive strategies or allocation of resources. The difference in incidence rates of nations and regions can depend on the accuracy of the detecting bodies in these nations to record SCI. Low incidence in sub-Saharan Africa and high age-standardised incidence rates in Greenland might be due to scarcity of data. Notably, some of the countries with the most substantial changes in age-standardised prevalence of SCI are those for which data are scarce or absent in GBD 2019. Greater resources might need to be invested in the collection and registration of data to improve the accuracy of future estimates.

Globally, incidence, prevalence, and YLDs were higher in males than in females throughout the period from 1990 to 2019, and the size of the difference between males and females remained almost stagnant for all the three indices, with a slight increase for both sexes during this period. Although the male-to-female ratio for SCI has been reported to vary in previous studies according to their methods, case definitions, and locations,^8^ males have always had higher rates of SCI than females. Men are usually more exposed to the causes of SCI, especially to the traumatic causes.^8^ Studies have shown that the male-to-female ratio of traumatic SCI decreases after age 54 years, suggesting that traumatic SCIs are more frequent among younger men than younger women, and they become progressively more relevant among women in later life, despite remaining more than twice as common among men.^27^ Moreover, GBD 2019 showed that males have divergent incidence patterns compared with women younger than 60 years but these patterns of change overlap in older age.^15^ In our study, however, 53·4% of the global incidence of SCI was in men in 2019, which showed a slight decrease in comparison with 1990 (57·0%). However, under-reporting of SCI among females due to non-inclusion of females in studies might be responsible for variation in injuries among males and females in 1990 as compared with 2019.^28^ There is an apparent reduction in global incidence of SCI between 1990 and 2019 among children and adolescents younger than 25 years. Whereas previously patients with SCI were thought to be relatively young,^8^ a shift towards older ages is seen in comparison with 1990, in addition to an increase in incidence rate. In our study, falls were the leading contributor to incidence numbers of SCI globally, followed by road injuries. However, this ranking might differ according to region, as a systematic review and meta-analysis by Golestani and colleagues emphasised that two leading causes of traumatic SCI in developing countries (based on the definition of developing countries by the International Monetary Fund) were motor vehicle crashes (43·2%) and falls (34·2%).^29^ A previous GBD subanalysis study also showed that transport injuries, one of the major causes of SCI, are more common in countries with a high SDI than in countries with a low SDI.^30^

WHO has estimated an annual incidence of SCI at between 250 000 to 500 000 people worldwide.^31^ However, depending on the region, the estimated incidences and prevalence can vary widely.^32^ van den Berg and colleagues, for instance, reported annual incidences of SCI from 1·2 cases per 100 000 population in the Netherlands to 5·8 cases per 100 000 population in Portugal.^24^ The annual incidence of SCI in developing countries (based on the definition of developing countries by the International Monetary Fund) yields an even wider range, from 0·2 cases per 100 000 population in Saudi Arabia up to 13·0 cases per 100 000 population in Bulgaria.^8^ This heterogeneity has been mainly rooted in diverse data-gathering methods, case-defining approaches, and socioeconomic structures of different countries.^9^ The global incidence of SCI in our study was more than 900 thousand cases in 2019 with a rate of 11·5 (95% UI 8·9–14·6) cases per 100 000 population. We believe that one main reason for the heterogeneity could be that most studies in this area on SCI focus on patients with traumatic SCI, and surveys of local and national incidences have been conducted in this context,^32^, ^33^ mostly neglecting patients with non-traumatic SCI. Furthermore, data access and quality are heterogeneous across different locations, and data in LMICs are particularly sparse. However, a few high-income countries, such as Australia, Canada, Switzerland, and the USA, have developed SCI systems^34^ that enable them to accurately monitor and periodically report epidemiological data. In some countries, many patients with SCI do not appear in the statistics because of the difficulty in assessing incidence, for example, owing to the absence of prehospital mortality data.

Efforts have been made to describe the true epidemiology of SCI in Iran,^35^ but there is still a strong need to understand epidemiological characteristics to promote practical preventive strategies in many other areas with poor data. Although single-centre series are being published from LMICs, some high-income European countries continue to update their knowledge periodically. However, the epidemiology of SCI varies in different countries and the results of studies from high-income countries are not applicable to LMICs.^36^, ^37^ In a systematic review for example, by use of an extensive search strategy, Jazayeri and colleagues identified 101 reports regarding traumatic SCI incidence up to 2013 from 41 countries, representing about 20% of countries worldwide.^34^ Moreover, most of the available literature evaluated the epidemiological aspects of SCI in high-income countries. In 2023, Jazayeri and colleagues updated their search strategy and found 58 new reports for 31 countries,^38^ implying that data compilation efforts are increasing globally. However, there is still a huge gap to fill to draw a more accurate global map for SCI incidence and prevalence, especially for LMICs. Another study using GBD 2019 data for SCI by Ding and colleagues was published in 2022.^39^ In comparison with their paper, which emphasises global and regional results with limited discussion of age and sex, we tried to provide a comprehensive display of global, regional, and national results as well as trends by sex and age, and we prepared our report with input from a network of global researchers as part of the GBD Protocol.

General limitations of GBD, which have been discussed previously,^40^ such as difficulties in accurately quantifying all sources of uncertainty, lags in data availability, heterogeneity in coding procedures, and other biases, fully apply to this report. GBD estimation of SCI is reconstructed by mathematical models using various sources of differing quality and is not a collection of measured data. This reconstruction might deviate from the actual data, particularly in some areas without adequate and reliable population-based data, such as sub-Saharan Africa or Greenland. These limitations might account for differences between these data and those collected by other governmental or disease-specific organisations. The quality of the data used in the predicting tool can also have an effect on the outcomes, which were mostly derived from the modelled data through the procedure in the DisMod-MR 2.1 tool. GBD 2019 estimates incidence, prevalence, mortality, years of life lost (YLLs), YLDs, and disability-adjusted life-years (DALYs) for 369 diseases and injuries. YLLs, which is the number of years lost due to premature death and represents a fatal burden, is not calculated by GBD 2019 for injuries categories and mortality data are not available; therefore DALYs, which are the sum of YLLs and YLDs, was not retrievable for SCI from the GBD 2019 database. The comparison of YLDs without consideration of socioeconomic factors might provide misleading data. However, presenting YLDs in our study could be considered an advantage over the GBD 2016 paper.^15^ Another methodological limitation is that data for the type of SCI (ie, traumatic *vs* non-traumatic), which can affect outcome substantially, are not currently retrievable from the GBD database. Traumatic SCI is abrupt and sudden, whereas non-traumatic SCI is usually progressive and gradual. This classification of data could be informative in many ways, including indicating age and sex differences. One other important issue regarding studies addressing SCI is differentiation between complete and incomplete lesions. There is a spectrum of clinical findings for SCI, which can be classified according to the American Spinal Injury Association score and Frankel grading. In other words, not all SCIs are permanent and associated with lost years of life and disability. People with minimal and mild injuries, and even some moderate injuries, can fully or near fully recover and return to healthy function and life. The nature of SCI affects the results and the burden of the disease significantly. Whereas incomplete SCIs are associated with better outcomes and less disability, complete lesions are permanent and carry substantial social, economic, and emotional burdens. Thus, these two entities should be separated when analysing and reporting the results of GBD. In future rounds of GBD, researchers should provide other epidemiological indices, such as YLL, DALYs, mortality, and life expectancy, for SCI so that the total (ie, fatal and non-fatal) burden of SCI can be provided, broken down by the cause of injury (ie, traumatic *vs* non-traumatic), and also type of the injury (ie, complete *vs* incomplete). The unavailability of data for SDI for SCI in the GBD database is another notable limitation of our study. Such data could have provided important information for economic and administrative planning purposes.

According to our results, epidemiology of SCI is increasingly affecting people at older ages, and the number of people affected is increasing globally. Although the age-standardised rates of incidence, prevalence, and YLDs showed only slight changes from 1990, the absolute counts of cases increased substantially. Our findings could aid health-care professionals and policy makers at the global or national levels in providing preventive interventions and advance planning for resource allocation to prevent and reduce the burden of SCI. Future studies should focus on considering the attributable risk factors and differences according to the severity and type of injuries, in addition to the potential effect of socioeconomic status on the burden of SCI.

## Data sharing

Data used in this article are available for download on the Global Health Data Exchange tool,^20^ which is permitted to be used, shared, modified, or built on by non-commercial users via the Open Data Commons Attribution License. All GBD 2019 data are publicly available and can be downloaded via the Global Health Data Exchange tool^20^ and from the GBD Compare Visualisation Tool.^41^

## Declaration of interests

R C Franklin reports grants or contracts from Heatwaves in Queensland—Queensland Government and Arc Flash–Human Factors–Queensland Government; payment or honoraria for lectures, presentations, speakers bureaus, manuscript writing, or educational events from Honoraria–World Safety Conference 2022–Conference Convener; support for attending meetings or travel from the Australasian College of Tropical Medicine for the Tropical Medicine and Travel Medicine Conference 2022 and from the International Society of Travel Medicine for the Travel Medicine Conference, Basel 2023; and leadership or fiduciary roles in board, society, committee, or advocacy groups, paid or unpaid with Kidsafe as Director, Farmsafe as Director, Auschem as Director, the Public Health Association of Australia Injury Prevention Special Interest Group as Convenor, and the International Society for Agricultural Safety and Health as a member of the Governance Committee, all outside the submitted work. E Trinka reports grants or contracts from the Austrian Science Fund (FWF), Oesterreichische Nationalbank, the EU, GSK, Biogen, Eisai, Novartis, Red Bull, Bayer, and UCB; consulting fees from Angelini, Clexio, Argenx, Arvelle, Epilog, Ever Pharma, UCB, Biogen, GSK, Bial, Eisai, Takeda, Newbridge, GW Pharma, Sunovion, Liva Nova, Marinus, Medtronic, Novartis, Sandoz, and Sanofi; payment or honoraria for lectures, presentations, speakers bureaus, manuscript writing, or educational events from UCB, Eisai, Biogen, Novartis, Bial, Sunovion, Ever Pharma, Liva Nova, Sanofi, Hikma, Newbridge, Arvelle, GW Pharma, and Sandoz; and other support from Neuroconsult as CEO, all outside the submitted work. All other authors declare no competing interests.
