# Correction to *Lancet Neurol* 2023; 22: 1026–47

**DOI:** 10.1016/S1474-4422(24)00095-4

**Published:** 2024-04

*GBD Spinal Cord Injuries Collaborators. Global, regional, and national burden of spinal cord injury, 1990–2019: a systematic analysis for the Global Burden of Disease Study 2019.* Lancet Neurol *2023; **22:** 1026–47*—In this Article, Ted R Miller should have been included in the GBD 2019 Spinal Injuries Collaborators list, and the appendix has been updated. This correction has been made to the online version as of March 18, 2024.

