# Supplementary material for: Global, regional, and national burden of spinal cord injury, 1990–2019: a systematic analysis for the Global Burden of Disease Study 2019
Source: Lancet Neurol. 2023 Nov;22(11):1026–47. doi: 10.1016/S1474-4422(23)00287-9 (PMC10584692; doi:10.1016/S1474-4422(23)00287-9)
Supplement: Supplementary appendix [file mmc1.pdf]

THE LANCET
Neurology

Supplementary appendix

This appendix formed part of the original submission and has been peer reviewed. We post it as supplied by the authors.

Supplement to: GBD Spinal Cord Injuries Collaborators. Global, regional, and national burden of spinal cord injury, 1990–2019: a systematic analysis for the Global Burden of Disease Study 2019. *Lancet Neurol* 2023; **22:** 1026–47.

Appendix

Global, regional, and national burden of spinal cord injury, 1990–2019: a systematic analysis for the Global Burden of Disease Study 2019

Content

Supplementary Table 1. International Classification of Diseases (ICD) codes list for spinal cord injury used in GBD modelling system……………………………………………………………………………………………………2

Supplementary table 2. Age-standardized rate per 100,000 population for incidence, prevalence, and years lived with disability of spinal cord injuries in GBD regions in 2019 and percent change from 1990-2019………7

Supplementary Table 3. Global numbers of incidence, prevalence and years lived with disability (×1000) for spinal cord injuries from all causes in 2019 according to age groups……………………………8

Supplementary Table 4. Global incidence, prevalence and years lived with disability (×1000) for spinal cord injuries from all causes and for all ages in 1990 and 2019, in addition to their age-standardized rate percent change separately for two sexes……………………………………………………………………………8

Supplementary Table 5. Global incidence, prevalence and years lived with disability and age-standardized rate per 100.000 population for spinal cord injuries in 2019, according to the cause of injury……9

Supplementary Figure 1. Global Burden of Disease (GBD) 21 Regions……………………………………………10

Supplementary Figure 2. Global trends of incidence, prevalence and years lived with disability of spinal cord injuries from 1990 to 2019 for male, female, and both sexes…………………………………………10

Supplementary Figure 3. Global incidence, prevalence and years lived with disability for spinal cord injuries from 1990 to 2019 from all causes and for both sexes and all ages according to the level of injury………11

Supplementary Figure 4. Global age-standardized rate (per 100,000-population) change of incidence, prevalence and years lived with disability for the two leading causes of spinal cord injuries (falls and road injuries) from 1990 to 2019……………………………………………………………………………………………11

Search strategies……12

Contributions to the paper………………………………………………………………………………………………………13

Supplementary Table 1. International Classification of Diseases (ICD) codes list for spinal cord injury used in GBD modelling system

Spinal lesions at neck level			
**ICD-9-CM**	**Definition**	**ICD-10 -CM**	**Definition**
**806**	Fracture of vertebral column with spinal cord injury	**S14**	Injury of nerves and spinal cord at neck level
**806.0**	Closed fracture of cervical vertebra with spinal cord injury	**S14.0**	Concussion and oedema of cervical spinal cord
**806.00**	Closed fracture of C1-C4 level with unspecified spinal cord injury	**S14.1**	Other and unspecified injuries of cervical spinal cord
**806.01**	Closed fracture of C1-C4 level with complete lesion of cord	**S14.10**	Unspecified injury of cervical spinal cord
**806.02**	Closed fracture of C1-C4 level with anterior cord syndrome	**S14.101**	Unspecified injury at C1 level of cervical spinal cord
**806.03**	Closed fracture of C1-C4 level with central cord syndrome	**S14.102**	Unspecified injury at C2 level of cervical spinal cord
**806.04**	Closed fracture of C1-C4 level with other specified spinal cord injury	**S14.103**	Unspecified injury at C3 level of cervical spinal cord
**806.05**	Closed fracture of C5-C7 level with unspecified spinal cord injury	**S14.104**	Unspecified injury at C4 level of cervical spinal cord
**806.06**	Closed fracture of C5-C7 level with complete lesion of cord	**S14.105**	Unspecified injury at C5 level of cervical spinal cord
**806.07**	Closed fracture of C5-C7 level with anterior cord syndrome	**S14.106**	Unspecified injury at C6 level of cervical spinal cord
**806.08**	Closed fracture of C5-C7 level with central cord syndrome	**S14.107**	Unspecified injury at C7 level of cervical spinal cord
**806.09**	Closed fracture of C5-C7 level with other specified spinal cord injury	**S14.108**	Unspecified injury at C8 level of cervical spinal cord
**806.1**	Open fracture of cervical vertebra with spinal cord injury	**S14.109**	Unspecified injury at unspecified level of cervical spinal cord
**806.10**	Open fracture of C1-C4 level with unspecified spinal cord injury	**S14.11**	Complete lesion of cervical spinal cord
**806.11**	Open fracture of C1-C4 level with complete lesion of cord	**S14.111**	Complete lesion at C1 level of cervical spinal cord
**806.12**	Open fracture of C1-C4 level with anterior cord syndrome	**S14.112**	Complete lesion at C2 level of cervical spinal cord
**806.13**	Open fracture of C1-C4 level with central cord syndrome	**S14.113**	Complete lesion at C3 level of cervical spinal cord
**806.14**	Open fracture of C1-C4 level with other specified spinal cord injury	**S14.114**	Complete lesion at C4 level of cervical spinal cord
**806.15**	Open fracture of C5-C7 level with unspecified spinal cord injury	**S14.115**	Complete lesion at C5 level of cervical spinal cord
**806.16**	Open fracture of C5-C7 level with complete lesion of cord	**S14.116**	Complete lesion at C6 level of cervical spinal cord
**806.17**	Open fracture of C5-C7 level with anterior cord syndrome	**S14.117**	Complete lesion at C7 level of cervical spinal cord
**806.18**	Open fracture of C5-C7 level with central cord syndrome	**S14.118**	Complete lesion at C8 level of cervical spinal cord
**806.19**	Open fracture of C5-C7 level with other specified spinal cord injury	**S14.119**	Complete lesion at unspecified level of cervical spinal cord

**952**	Spinal cord injury without evidence of spinal bone injury	**S14.12**	Central cord syndrome of cervical spinal cord
**952.0**	Cervical spinal cord injury without evidence of spinal bone injury	**S14.121**	Central cord syndrome at C1 level of cervical spinal cord
**952.00**	C1-C4 level with unspecified spinal cord injury	**S14.122**	Central cord syndrome at C2 level of cervical spinal cord
**952.01**	C1-C4 level with complete lesion of spinal cord	**S14.123**	Central cord syndrome at C3 level of cervical spinal cord
**952.02**	C1-C4 level with anterior cord syndrome	**S14.124**	Central cord syndrome at C4 level of cervical spinal cord
**952.03**	C1-C4 level with central cord syndrome	**S14.125**	Central cord syndrome at C5 level of cervical spinal cord
**952.04**	C1-C4 level with other specified spinal cord injury	**S14.126**	Central cord syndrome at C6 level of cervical spinal cord
**952.05**	C5-C7 level with unspecified spinal cord injury	**S14.127**	Central cord syndrome at C7 level of cervical spinal cord
**952.06**	C5-C7 level with complete lesion of spinal cord	**S14.128**	Central cord syndrome at C8 level of cervical spinal cord
**952.07**	C5-C7 level with anterior cord syndrome	**S14.129**	Central cord syndrome at unspecified level of cervical spinal cord
**952.08**	C5-C7 level with central cord syndrome	**S14.13**	Anterior cord syndrome of cervical spinal cord
**952.09**	C5-C7 level with other specified spinal cord injury	**S14.131**	Anterior cord syndrome at C1 level of cervical spinal cord
		**S14.132**	Anterior cord syndrome at C2 level of cervical spinal cord
		**S14.133**	Anterior cord syndrome at C3 level of cervical spinal cord
		**S14.134**	Anterior cord syndrome at C4 level of cervical spinal cord
		**S14.135**	Anterior cord syndrome at C5 level of cervical spinal cord
		**S14.136**	Anterior cord syndrome at C6 level of cervical spinal cord
		**S14.137**	Anterior cord syndrome at C7 level of cervical spinal cord
		**S14.138**	Anterior cord syndrome at C8 level of cervical spinal cord
		**S14.139**	Anterior cord syndrome at unspecified level of cervical spinal cord
		**S14.14**	Brown-Séquard syndrome of cervical spinal cord
		**S14.141**	Brown-Séquard syndrome at C1 level of cervical spinal cord
		**S14.142**	Brown-Séquard syndrome at C2 level of cervical spinal cord
		**S14.143**	Brown-Séquard syndrome at C3 level of cervical spinal cord
		**S14.144**	Brown-Séquard syndrome at C4 level of cervical spinal cord
		**S14.145**	Brown-Séquard syndrome at C5 level of cervical spinal cord
		**S14.146**	Brown-Séquard syndrome at C6 level of cervical spinal cord

		**S14.147**	Brown-Séquard syndrome at C7 level of cervical spinal cord
		**S14.148**	Brown-Séquard syndrome at C8 level of cervical spinal cord
		**S14.149**	Brown-Séquard syndrome at unspecified level of cervical spinal cord
		**S14.15**	Other incomplete lesions of cervical spinal cord
		**S14.151**	Other incomplete lesion at C1 level of cervical spinal cord
		**S14.152**	Other incomplete lesion at C2 level of cervical spinal cord
		**S14.153**	Other incomplete lesion at C3 level of cervical spinal cord
		**S14.154**	Other incomplete lesion at C4 level of cervical spinal cord
		**S14.155**	Other incomplete lesion at C5 level of cervical spinal cord
		**S14.156**	Other incomplete lesion at C6 level of cervical spinal cord
		**S14.157**	Other incomplete lesion at C7 level of cervical spinal cord
		**S14.158**	Other incomplete lesion at C8 level of cervical spinal cord
		**S14.159**	Other incomplete lesion at unspecified level of cervical spinal cord
		**T91.3**	Sequelae of injury of spinal cord
**Spinal lesions below neck level**			
**ICD-9-CM**	**Definition**	**ICD-10-CM**	**Definition**
**806.2**	Closed fracture of dorsal vertebra with spinal cord injury	**S24**	Injury of nerves and spinal cord at thorax level
**806.20**	Closed fracture of T1-T6 level with unspecified spinal cord injury	**S24.0**	Concussion and edema of thoracic spinal cord
**806.21**	Closed fracture of T1-T6 level with complete lesion of cord	**S24.1**	Other and unspecified injuries of thoracic spinal cord
**806.22**	Closed fracture of T1-T6 level with anterior cord syndrome	**S24.10**	Unspecified injury of thoracic spinal cord
**806.23**	Closed fracture of T1-T6 level with central cord syndrome	**S24.101**	Unspecified injury at T1 level of thoracic spinal cord
**806.24**	Closed fracture of T1-T6 level with other specified spinal cord injury	**S24.102**	Unspecified injury at T2-T6 level of thoracic spinal cord
**806.25**	Closed fracture of T7-T12 level with unspecified spinal cord injury	**S24.103**	Unspecified injury at T7-T10 level of thoracic spinal cord
**806.26**	Closed fracture of T7-T12 level with complete lesion of cord	**S24.104**	Unspecified injury at T11-T12 level of thoracic spinal cord
**806.27**	Closed fracture of T7-T12 level with anterior cord syndrome	**S24.109**	Unspecified injury at unspecified level of thoracic spinal cord
**806.28**	Closed fracture of T7-T12 level with central cord syndrome	**S24.11**	Complete lesion of thoracic spinal cord
**806.29**	Closed fracture of T7-T12 level with other specified spinal cord injury	**S24.111**	Complete lesion at T1 level of thoracic spinal cord

**806.3**	Open fracture of dorsal vertebra with spinal cord injury	**S24.112**	Complete lesion at T2-T6 level of thoracic spinal cord
**806.30**	Open fracture of T1-T6 level with unspecified spinal cord injury	**S24.113**	Complete lesion at T7-T10 level of thoracic spinal cord
**806.31**	Open fracture of T1-T6 level with complete lesion of cord	**S24.114**	Complete lesion at T11-T12 level of thoracic spinal cord
**806.32**	Open fracture of T1-T6 level with anterior cord syndrome	**S24.119**	Complete lesion at unspecified level of thoracic spinal cord
**806.33**	Open fracture of T1-T6 level with central cord syndrome	**S24.13**	Anterior cord syndrome of thoracic spinal cord
**806.34**	Open fracture of T1-T6 level with other specified spinal cord injury	**S24.131**	Anterior cord syndrome at T1 level of thoracic spinal cord
**806.35**	Open fracture of T7-T12 level with unspecified spinal cord injury	**S24.132**	Anterior cord syndrome at T2-T6 level of thoracic spinal cord
**806.36**	Open fracture of T7-T12 level with complete lesion of cord	**S24.133**	Anterior cord syndrome at T7-T10 level of thoracic spinal cord
**806.37**	Open fracture of T7-T12 level with anterior cord syndrome	**S24.134**	Anterior cord syndrome at T11-T12 level of thoracic spinal cord
**806.38**	Open fracture of T7-T12 level with central cord syndrome	**S24.139**	Anterior cord syndrome at unspecified level of thoracic spinal cord
**806.39**	Open fracture of T7-T12 level with other specified spinal cord injury	**S24.14**	Brown-Séquard syndrome of thoracic spinal cord
**806.4**	Closed fracture of lumbar spine with spinal cord injury	**S24.141**	Brown-Séquard syndrome at T1 level of thoracic spinal cord
**806.5**	Open fracture of lumbar spine with spinal cord injury	**S24.142**	Brown-Séquard syndrome at T2-T6 level of thoracic spinal cord
**806.6**	Closed fracture of sacrum and coccyx with spinal cord injury	**S24.143**	Brown-Séquard syndrome at T7-T10 level of thoracic spinal cord
**806.60**	Closed fracture of sacrum and coccyx with unspecified spinal cord injury	**S24.144**	Brown-Séquard syndrome at T11-T12 level of thoracic spinal cord
**806.61**	Closed fracture of sacrum and coccyx with complete cauda equina lesion	**S24.149**	Brown-Séquard syndrome at unspecified level of thoracic spinal cord
**806.62**	Closed fracture of sacrum and coccyx with other cauda equina injury	**S24.15**	Other incomplete lesions of thoracic spinal cord
**806.69**	Closed fracture of sacrum and coccyx with other spinal cord injury	**S24.151**	Other incomplete lesion at T1 level of thoracic spinal cord
**806.7**	Open fracture of sacrum and coccyx with spinal cord injury	**S24.152**	Other incomplete lesion at T2-T6 level of thoracic spinal cord
**806.70**	Open fracture of sacrum and coccyx with unspecified spinal cord injury	**S24.153**	Other incomplete lesion at T7-T10 level of thoracic spinal cord
**806.71**	Open fracture of sacrum and coccyx with complete cauda equina lesion	**S24.154**	Other incomplete lesion at T11-T12 level of thoracic spinal cord
**806.72**	Open fracture of sacrum and coccyx with other cauda equina injury	**S24.159**	Other incomplete lesion at unspecified level of thoracic spinal cord
**806.79**	Open fracture of sacrum and coccyx with other spinal cord injury	**S34**	Injury of lumbar and sacral spinal cord and nerves at abdomen, lower back and pelvis level
**806.8**	Closed fracture of unspecified vertebral column with spinal cord injury	**S34.0**	Concussion and edema of lumbar and sacral spinal cord
**806.9**	Open fracture of unspecified vertebral column with spinal cord injury	**S34.01**	Concussion and edema of lumbar spinal cord
**952.1**	Dorsal (thoracic) spinal cord injury without evidence of spinal bone injury	**S34.02**	Concussion and edema of sacral spinal cord

**952.10**	T1-T6 level with unspecified spinal cord injury	**S34.1**	Other and unspecified injury of lumbar and sacral spinal cord
**952.11**	T1-T6 level with complete lesion of spinal cord	**S34.10**	Unspecified injury to lumbar spinal cord
**952.12**	T1-T6 level with anterior cord syndrome	**S34.101**	Unspecified injury to L1 level of lumbar spinal cord
**952.13**	T1-T6 level with central cord syndrome	**S34.102**	Unspecified injury to L2 level of lumbar spinal cord
**952.14**	T1-T6 level with other specified spinal cord injury	**S34.103**	Unspecified injury to L3 level of lumbar spinal cord
**952.15**	T7-T12 level with unspecified spinal cord injury	**S34.104**	Unspecified injury to L4 level of lumbar spinal cord
**952.16**	T7-T12 level with complete lesion of spinal cord	**S34.105**	Unspecified injury to L5 level of lumbar spinal cord
**952.17**	T7-T12 level with anterior cord syndrome	**S34.109**	Unspecified injury to unspecified level of lumbar spinal cord
**952.18**	T7-T12 level with central cord syndrome	**S34.11**	Complete lesion of lumbar spinal cord
**952.19**	T7-T12 level with other specified spinal cord injury	**S34.111**	Complete lesion of L1 level of lumbar spinal cord
**952.2**	Lumbar spinal cord injury without evidence of spinal bone injury	**S34.112**	Complete lesion of L2 level of lumbar spinal cord
**952.3**	Sacral spinal cord injury without evidence of spinal bone injury	**S34.113**	Complete lesion of L3 level of lumbar spinal cord
**952.4**	Cauda equina spinal cord injury without evidence of spinal bone injury	**S34.114**	Complete lesion of L4 level of lumbar spinal cord
**952.8**	Multiple sites of spinal cord injury without evidence of spinal bone injury	**S34.115**	Complete lesion of L5 level of lumbar spinal cord
**952.9**	Unspecified site of spinal cord injury without evidence of spinal bone injury	**S34.119**	Complete lesion of unspecified level of lumbar spinal cord
		**S34.12**	Incomplete lesion of lumbar spinal cord
		**S34.121**	Incomplete lesion of L1 level of lumbar spinal cord
		**S34.122**	Incomplete lesion of L2 level of lumbar spinal cord
		**S34.123**	Incomplete lesion of L3 level of lumbar spinal cord
		**S34.124**	Incomplete lesion of L4 level of lumbar spinal cord
		**S34.125**	Incomplete lesion of L5 level of lumbar spinal cord
		**S34.129**	Incomplete lesion of unspecified level of lumbar spinal cord
		**S34.13**	Other and unspecified injury to sacral spinal cord
		**S34.131**	Complete lesion of sacral spinal cord
		**S34.132**	Incomplete lesion of sacral spinal cord
		**S34.139**	Unspecified injury to sacral spinal cord

Supplementary table 2. Age-standardized rate per 100,000 population for incidence, prevalence, and years lived with disability of spinal cord injuries in GBD regions in 2019 and percent change from 1990-2019

	Incidence		Prevalence		Years lived with disability	
	2019	1990-2019 Change %	2019	1990-2019 Change %	2019	1990-2019 Change %
**Global**	**11 (9 to 15)**	**-6.1 (-17.2 to 1.5)**	**253 (231 to 290)**	**5.8 (2.6 to 9.5)**	**76 (55 to 100)**	**-1.5 (-5.5 to 3.2)**
**Andean Latin America**	4 (3-5)	-39.5 (-66.5 to -11.1)	135 (120-169)	-1.2 (-13.1 to 7.9)	43 (30-61)	-17.2 (-29.4 to -6.1)
**Australasia**	14 (11-17)	8.1 (3.3 to 13.2)	362 (331-405)	6.8 (3.9 to 9.8)	96 (67-127)	6.6 (0.7 to 13.0)
**Caribbean**	7 (5-8)	8.1 (0.9 to 15.1)	239 (187-336)	64.7 (29.1 to 131.2)	77 (49-117)	66.0 (24.3 to 140.0)
**Central Asia**	6 (5-8)	-6.8 (-9.5 to -4.3)	185 (169-212)	-2.7 (-9.7 to 10.6)	58 (41-76)	-8.0 (-16.6 to 8.0)
**Central Europe**	13 (10-15)	-12.9 (-16.1 to -9.8)	332 (308-363)	1.7 (-1.3 to 6.8)	91 (65-119)	-8.3 (-13.0 to -2.0)
**Central Latin America**	10 (7-12)	-23.4 (-34.2 to -17.9)	258 (234-299)	-16.7 (-24.4 to -11.9)	78 (56-103)	-28.1 (-36.4 to -22.5)
**Central Sub-Saharan Africa**	4 (3-6)	-29.1 (-47.9 to -10.3)	153 (92-312)	30.3 (16.9 to 44.5)	62 (33-134)	25.2 (8.7 to 40.0)
**East Asia**	13 (9-18)	39.8 (32.0 to 48.0)	266 (248-287)	37.9 (34.0 to 42.3)	72 (50-93)	11.1 (4.9 to 16.8)
**Eastern Europe**	13 (10-16)	-15.6 (-18.3 to -12.9)	305 (282-333)	-13.8 (-16.4 to -9.0)	84 (59-109)	-21.3 (-25 to -15.3)
**Eastern Sub-Saharan Africa**	8 (6-11)	-69.7 (-86.8 to -36.7)	234 (178-376)	8.3 (-3.2 to 15.3)	90 (57-160)	1.0 (-10.3 to 8.8)
**High-income Asia Pacific**	11 (9-14)	-9.7 (-13.9 to -6.4)	304 (283-328)	-3.9 (-5.7 to -1.8)	82 (57-106)	-5.3 (-7.9 to -2.6)
**High-income North America**	22 (17-29)	4.5 (-0.9 to 10.5)	437 (404-474)	-6.4 (-9.1 to -3.7)	113 (80-146)	-7.6 (-10.4 to -4.8)
**North Africa and Middle East**	9 (5-17)	-32.6 (-60.7 to 8.9)	264 (165-520)	5.0 (-19.4 to 21.3)	92 (47-198)	-5.4 (-27.6 to 12.3)
**Oceania**	5 (4-6)	10.2 (-2.6 to 18.9)	103 (95-116)	25.9 (19.5 to 36.0)	36 (26-46)	21.8 (9.5 to 35.9)
**South Asia**	9 (7-12)	5.0 (-7.7 to 12.7)	187 (175-202)	22.8 (19.4 to 27.0)	63 (46-79)	11.8 (7.6 to 16.9)
**Southeast Asia**	7 (5-9)	-18.6 (-31.8 to -10.7)	178 (161-206)	1.2 (-10.6 to 9.3)	58 (42-77)	-9.2 (-22.3 to -0.6)
**Southern Latin America**	7 (6-9)	1.7 (-2.5 to 5.1)	208 (194224)	7.7 (0.4 to 12.2)	60 (42-77)	-4.8 (-14 to 2.9)
**Southern Sub-Saharan Africa**	8 (6-11)	-11.9 (-16.4 to -8.3)	170 (159-183)	-20.3 (-25.3 to -17.5)	56 (40-70)	-25.2 (-30.8 to -21.5)
**Tropical Latin America**	14 (10-19)	-8.1 (-11.5 to -5.0)	324 (299-354)	-2.5 (-5.4 to 0.3)	96 (69-123)	-12.8 (-16.6 to -9)
**Western Europe**	9 (7-12)	-5.6 (-9.8 to -2.1)	232 (215-252)	1.4 (-1.4 to 4.3)	62 (44-81)	1.3 (-2.2 to 5.1)
**Western Sub-Saharan Africa**	11 (8-15)	3.3 (-5.4 to 7.9)	215 (197-241)	17.7 (14.0 to 26.0)	75 (54-97)	12.7 (7.8 to 22.6)

Supplementary Table 3. Global numbers of incidence, prevalence and years lived with disability (×1000) for spinal cord injuries from all causes in 2019 according to age groups

Age	Incidence	Prevalence	Years Lived with Disability
**<1 year**	6 (4-11)	2 (1.7-2.4)	0.8 (0.6-1.1)
**1 to 4**	20 (15-29)	69 (52-108)	26 (17-44)
**5 to 9**	28 (21-38)	237 (184-355)	87 (54-146)
**10 to 14**	35 (26-47)	418 (341-566)	148 (97-224)
**15 to 19**	45 (32-62)	634 (538-815)	220 (148-317)
**20 to 24**	53 (39-74)	922 (796-1,158)	316 (215-449)
**25 to 29**	56 (40-77)	1,246 (1,091-1,529)	416 (287-580)
**30 to 34**	57 (41-81)	1,590 (1,398-1,945)	520 (368-714)
**35 to 39**	54 (39-73)	1,739 (1,520-2,162)	563 (390-783)
**40 to 44**	51 (37-71)	1,806 (1,621-2,151)	571 (403-779)
**45 to 49**	55 (39-76)	1,944 (1,768-2,249)	596 (425-783)
**50 to 54**	58 (41-80)	1,979 (1,811-2,224)	586 (418-764)
**55 to 59**	56 (39-77)	1,791 (1,654-1,979)	512 (368-664)
**60 to 64**	55 (38-79)	1,631 (1,505-1,782)	451 (324-581)
**65 to 69**	57 (39-81)	1,503 (1,369-1,642)	404 (293-516)
**70 to 74**	53 (35-78)	1,175 (1,054-1,300)	306 (222-392)
**75 to 79**	51 (33-79)	858 (746-988)	217 (157-284)
**80 to 84**	51 (33-77)	596 (492-755)	145 (101-195)
**85 to 89**	37 (24-59)	325 (253-420)	76 (51-105)
**90 to 94**	20 (13-31)	132 (92-177)	29 (19-43)
**95 plus**	8 (4-13)	36 (24-53)	7 (5-12)

Supplementary Table 4. Global incidence, prevalence and years lived with disability (×1000) for spinal cord injuries from all causes and for all ages in 1990 and 2019, in addition to their age-standardized rate percent change separately for two sexes

		1990	2019	1990-2019 age-standardized rate change %
**Incidence**	**Male**	339 (269-434)	485 (386-609)	-7.7 (-18.9–0.1)
	**Female**	256 (201-336)	423 (321-555)	-3.2 (-13.9–3.9)
**Prevalence**	**Male**	6,394 (5,833-7,360)	11,455 (10,505-12,999)	6.4 (3–9.5)
	**Female**	4,967 (4,532-5,801)	9,180 (8,400-10,668)	5.8 (2.8–10.3)
**Years lived with disability**	**Male**	2,159 (1,540-2,822)	3,526 (2,519-4,604)	-1.2 (-5.7–2.9)
	**Female**	1,589 (1,146-2,134)	2,674 (1,921-3,583)	-1.3 (-5.4–4.3)

Supplementary Table 5. Global incidence, prevalence and years lived with disability and age-standardized rate per 100.000 population for spinal cord injuries according to the cause of injury in 2019.

Causes of injury	Incidence		Prevalence		Years lived with disability	
	number	age-standardized rate	number	age-standardized rate	number	age-standardized rate
**Transport injuries**	**252 (142-412)**	**3.3 (1.8-5.3)**	**6345 (5768-6932)**	**82 (74.5-89.6)**	**1921 (1375-2461)**	**24.8 (17.8-31.8)**
Road injuries	230 (122-389)	3 (1.6-5)	5776 (5226-6372)	74.6 (67.5-82.4)	1755 (1244-2260)	22.7 (16.1-29.2)
Other transport injuries	22 (14-33)	0.3 (0.2-0.4)	569 (484-669)	7.3 (6.3-8.6)	166 (116-222)	2.2 (1.5-2.9)
**Unintentional injuries**	**584 (431-791)**	**7.5 (5.6-10.2)**	**11479 (10506-12589)**	**148.4 (135.8-162.7)**	**3275 (2298-4212)**	**42.3 (29.7-54.4)**
Falls	477 (327-683)	6.2 (4.2-8.8)	8450 (7519-9460)	109.2 (97.2-122.3)	2380 (1670-3104)	30.8 (21.6-40.1)
Drowning	3 (1-5)	0 (0-0.1)	69 (55-82)	0.9 (0.7-1.1)	21 (14-28)	0.3 (0.2-0.4)
Fire, heat, and hot substances	5 (3-8)	0.1 (0-0.1)	151 (131-173)	1.9 (1.7-2.2)	43 (31-57)	0.6 (0.4-0.7)
Poisonings	1 (0-2)	0 (0-0)	48 (41-56)	0.6 (0.5-0.7)	14 (10-19)	0.2 (0.1-0.2)
Exposure to mechanical forces	27 (13-50)	0.4 (0.2-0.6)	886 (764-1055)	11.5 (9.9-13.6)	259 (179-348)	3.4 (2.3-4.5)
Animal contact	15 (6-29)	0.2 (0.1-0.4)	1 (0-2)	0 (0-0)	1 (0-2)	0 (0-0)
Foreign body	27 (18-41)	0.4 (0.2-0.5)	718 (607-858)	9.3 (7.8-11.1)	216 (152-286)	2.8 (2-3.7)
Environmental heat and cold exposure	6 (3-11)	0.1 (0-0.1)	195 (161-231)	2.5 (2.1-3)	61 (41-82)	0.8 (0.5-1.1)
Exposure to forces of nature	0 (0-1)	0 (0-0)	330 (145-660)	4.3 (1.9-8.5)	104 (42-213)	1.3 (0.5-2.8)
Other unintentional injuries	22 (13-33)	0.3 (0.2-0.4)	630 (531-733)	8.1 (6.9-9.5)	176 (121-234)	2.3 (1.6-3)
**Self-harm and interpersonal violence**	**73 (43-131)**	**0.9 (0.6-1.7)**	**2811 (1661-5742)**	**36.3 (21.5-74.2)**	**1004 (498-2302)**	**13 (6.4-29.8)**
Self-harm	14 (6-27)	0.2 (0.1-0.4)	447 (355-566)	5.8 (4.6-7.3)	129 (84-179)	1.7 (1.1-2.3)
Interpersonal violence	32 (16-58)	0.4 (0.2-0.8)	931 (794-1097)	12 (10.3-14.2)	279 (194-368)	3.6 (2.5-4.8)
Conflict and terrorism	26 (6-81)	0.3 (0.1-1.1)	1400 (307-4381)	18.1 (4-56.6)	585 (129-1849)	7.6 (1.7-23.9)
Executions and police conflict	1 (0-2)	0 (0-0)	33 (14-70)	0.4 (0.2-0.9)	11 (4-25)	0.1 (0.1-0.3)

Supplementary figure 1. Global Burden of Disease (GBD) 21 Regions

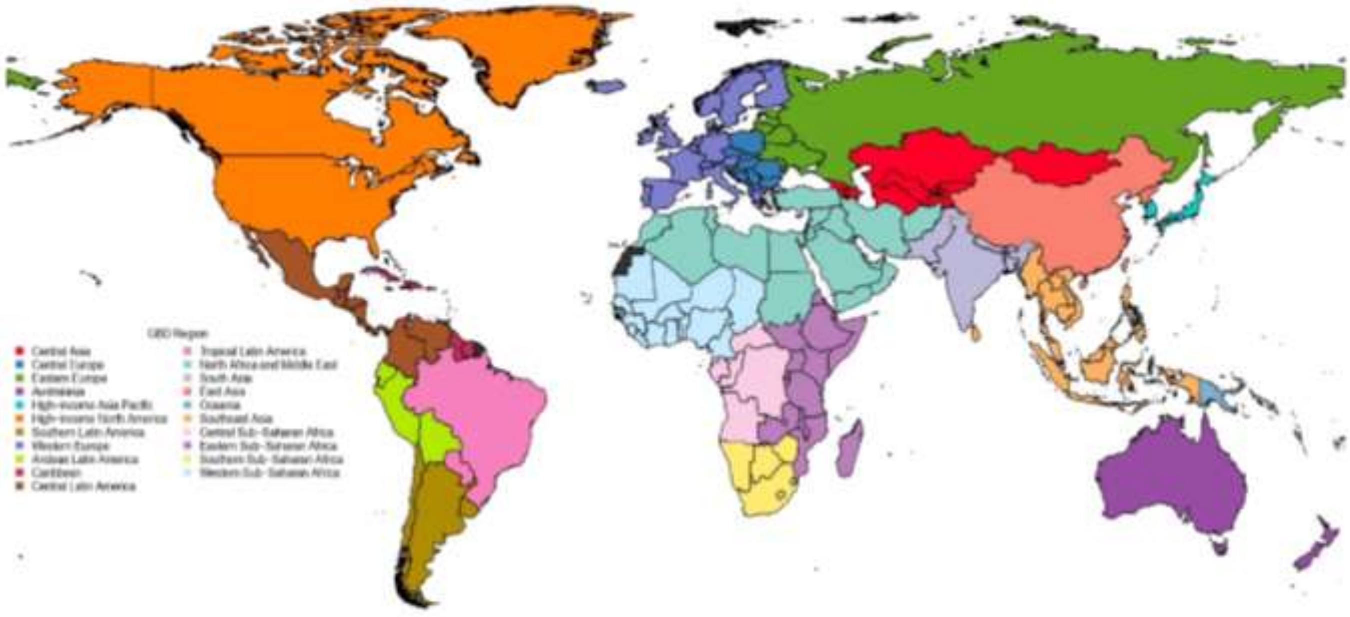

Supplementary Figure 2. Global trends of incidence, prevalence and years lived with disability of spinal cord injuries from 1990 to 2019 for male, female, and both sexes (95% UI are available in supplementary table 4)

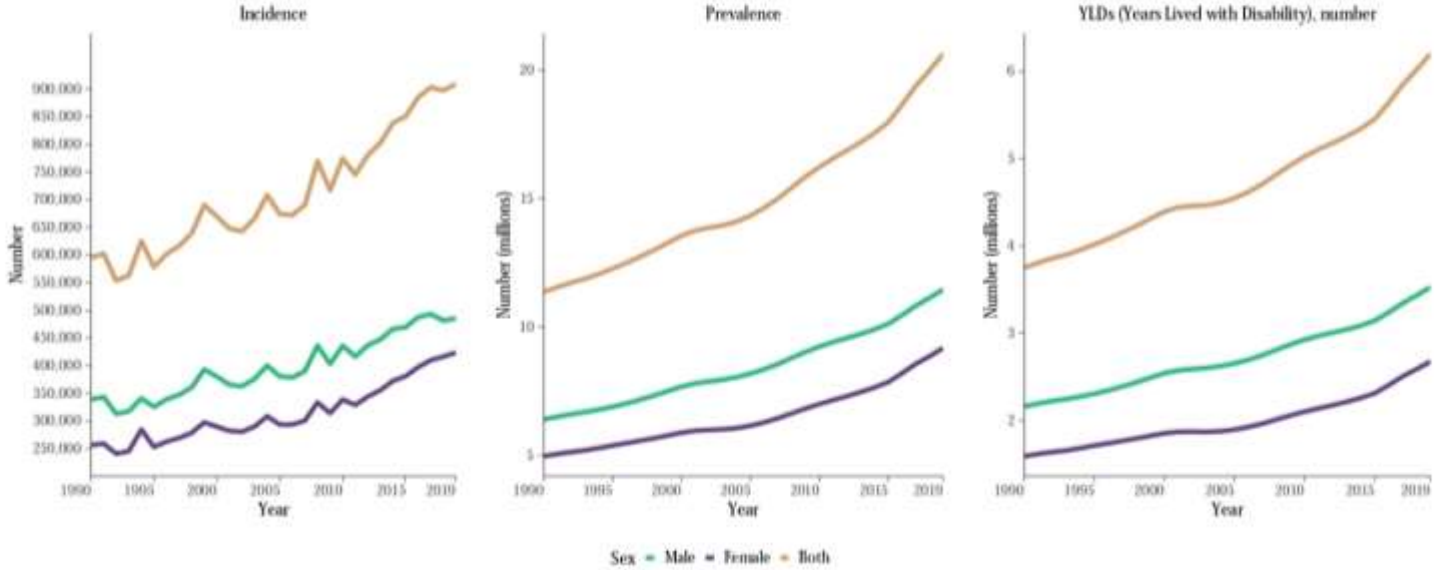

Supplementary Figure 3. Global incidence, prevalence and years lived with disability for spinal cord injuries from 1990 to 2019 from all causes and for both sexes and all ages according to the level of injury

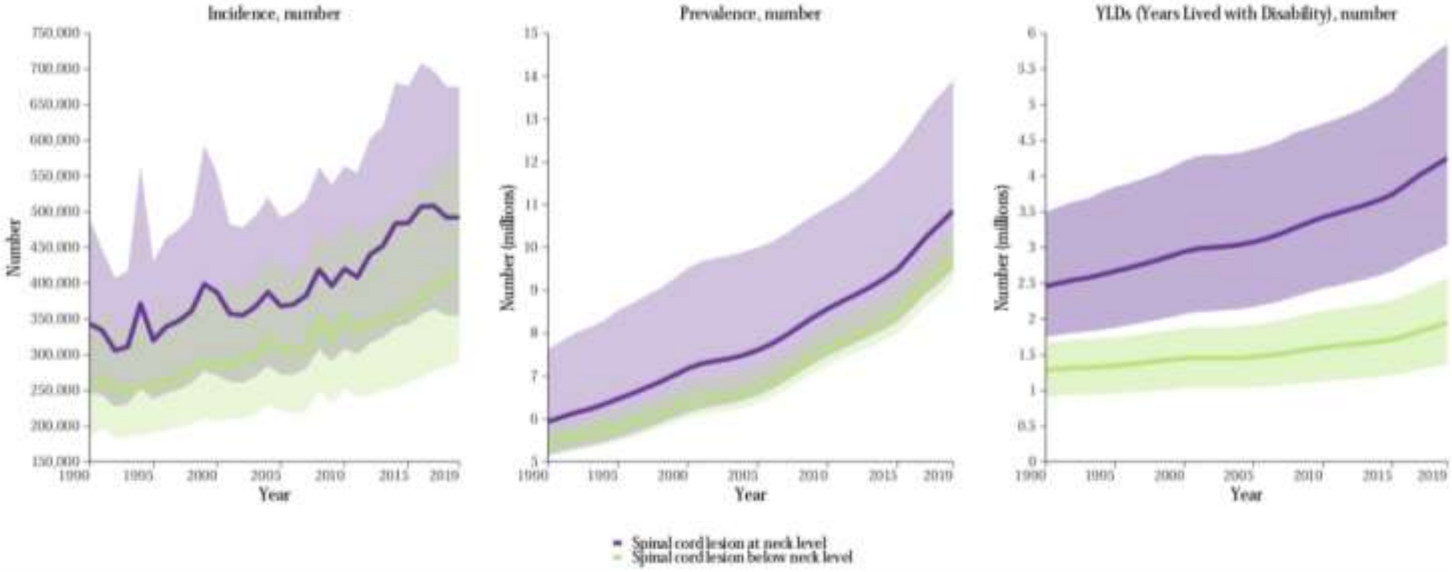

Supplementary Figure 4. Global age-standardized rate (per 100,000-population) change of incidence, prevalence and years lived with disability for the two leading causes of spinal cord injuries (falls and road injuries) from 1990 to 2019 (95% UI are shown in shaded purple)

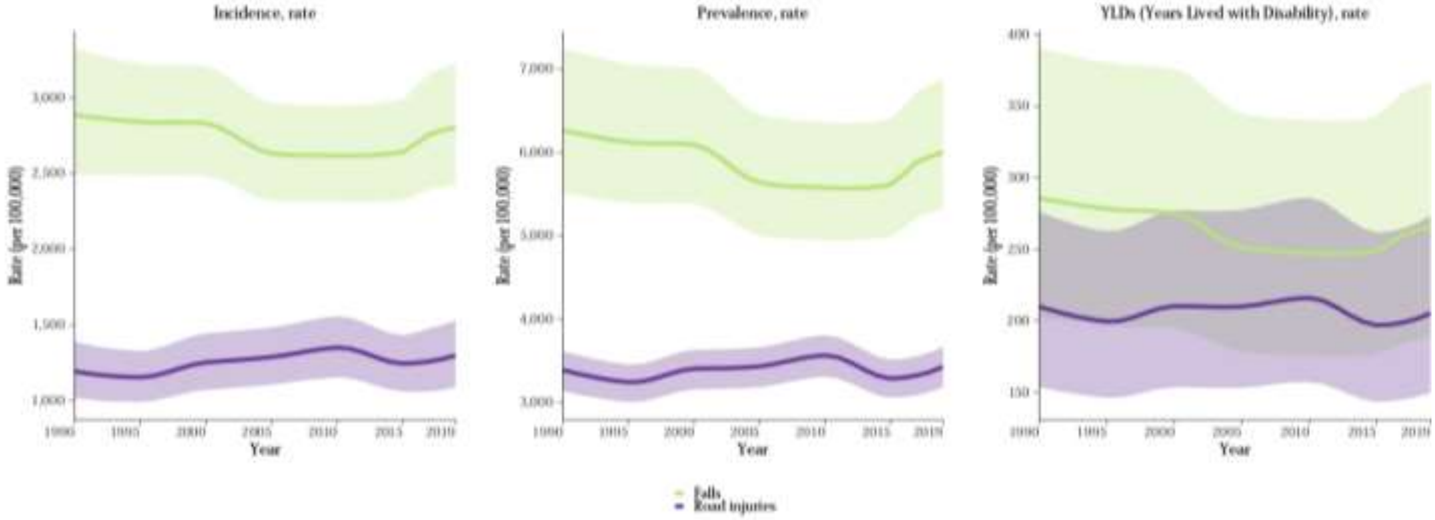

Search strategies

Search Strategy for Pubmed [2022/07/22]

#1,"Search (((spinal[title] AND cord[title/abstract] AND injur*[title/abstract])))",24948,15:10:05

#2,"Search (((epidemiolog*[Title/Abstract]) OR prevalence[Title/Abstract]) OR incidence[Title/Abstract)))",1203736,15:10:22

#3,"Search (((#1 AND #2)))",1758,15:10:3

#4,"Search (((#1 AND #2))) Filters: Humans",1451,15:10:43

#5,"Search (((((((spinal[title] AND cord[title/abstract] AND injur*[title/abstract]))))) AND ((((epidemiolog*[Title/Abstract]) OR prevalence[Title/Abstract]) OR incidence[Title/Abstract))))) AND ((((#1 AND #2))))) AND ((((#1 AND #2))) AND Humans[Mesh])) AND (""2012/07/29""[Date - Entrez] : ""3000""[Date - Entrez]) Filters: Humans",240,15:15:02

Final: ''(spinal[title/abstract] AND cord[title/abstract] AND injur*[title/abstract]) AND (epidemiolog*[title/abstract] OR prevalence[title/abstract] OR incidence[title/abstract])''

Search Strategy for Medline [2022/07/22] via Ovid SP

#1 spinal.ti. , 102721

#2 cord.ti,ab. , 167147

#3 injur$.ti,ab , 590595

#4 1 and 2 and 3 , 24229
#5 (epidemiolog$ or prevalence or incidence).ti,ab. , 1166270

#6 4 and 5 , 1701

#7 limit 6 to human , 1453

#8 limit 7 to ed=20120729-20160222 , 282

Search Strategy for Embase [2022/07/22]

#1 spinal:ti ,121577

#2 cord:ti,ab , 212449

#3 injur*:ti,ab , 753122

#4 #1 AND #2 AND #3 , 30182

#5 epidemiolog*:ab,ti OR prevalence:ab,ti OR incidence:ab,ti , 1554165

#6 #4 AND #5 , 2209

#7 #5 AND #6 AND [humans]/lim , 1925

#8 #9 AND [29-7-2012]/sd NOT [23-2-2016]/sd , 592

Contributions to the paper

Providing data or critical feedback on data sources

Semagn Mekonnen Abate, Aidin Abedi, Denberu Eshetie Adane, Saira Afzal, Bright Opoku Ahinkorah, Sajjad Ahmad, Haroon Ahmed, Nasir Amanat, Dhanalakshmi Angappan, Jalal Arabloo, Seyyed Shamsadin Athari, Alok Atreya, Ahmed Y Azzam, Palash Chandra Banik, Mainak Bardhan, Ajay Nagesh Bhat, Periklis Charalampous, Xiaochen Dai, Lalit Dandona, Rakhi Dandona, Fikadu Nugusu Dessalegn, Haneil Larson Dsouza, Michael Ekholuenetale, Adeniyi Francis Fagbamigbe, Jawad Fares, Ali Fatehizadeh, Seyed-Mohammad Fereshtehnejad, Richard Charles Franklin, Tushar Garg, Melaku Getachew, Fariborz Ghaffarpasand, Milad Gholizadeh Mesgarha, Sherief Ghozy, Mahaveer Golechha, Pouya Goleij, Vivek Kumar Gupta, Juanita A. Haagsma, Netanja I. Harlianto, Amir Human Hoveidaei, Farideh Iravanpour, Rana Irilouzadian, Chidozie C D Iwu, Charity Ehimwenma Joshua, Jacek Jerzy Jozwiak, Vidya Kadashetti, Rami S. Kantar, Samad Karkhah, Yousef Saleh Khader, Mohammad Saeid Khonji, Grace Kim, Vijay Krishnamoorthy, Senthil D Kumaran, Mohamed Kamal Mesregah, Awoke Misganaw, Ali H Mokdad, Sara Momtazmanesh, Ebrahim Mostafavi, Francesk Mulita, Mohsen Naghavi, Kazem Nejati, Huong Lan Thi Nguyen, Van Thanh Nguyen, Antonio Tolentino Nogueira de Sá, Andrew T Olagunju, Abiodun Olusola Omotayo, Mayowa O Owolabi, Shankargouda Patil, Shrikant Pawar, Vafa Rahimi-Movaghar, Salman Rawaf, Nicholas L S Roberts, Basema Saddik, Umar Saeed, Mahdi Safdarian, Sara Samadzadeh, Abdallah M Samy, Arash Sarveazad, Allen Seylani, Mequannent Melaku Sharew Sharew, Parnian Shobeiri, Yonatan Solomon, Houman Sotoudeh, Belsti Atnkut Tadesse, Sahel Valadan Tahbaz, Pascual R Valdez, Narayanaswamy Venketasubramanian, Linh Gia Vu, Dereje Y Yada, Iman Zare, Zhi-Jiang Zhang.

Developing methods or computational machinery

Saira Afzal, Nasir Amanat, Ahmed Y Azzam, Kaleb Coberly, Xiaochen Dai, Fikadu Nugusu Dessalegn, Ali Fatehizadeh, Melaku Getachew, Sherief Ghozy, Farideh Iravanpour, Samad Karkhah, Ali H Mokdad, Francesk Mulita, Mohsen Naghavi, Huong Lan Thi Nguyen, Van Thanh Nguyen, Abiodun Olusola Omotayo, Nicholas L S Roberts, Umar Saeed, Mahdi Safdarian, Abdallah M Samy, Linh Gia Vu.

Providing critical feedback on methods or results

Amirali Aali, Gdiom Gebreheat Abady, Aidin Abedi, Denberu Eshetie Adane, Saira Afzal, Bright Opoku Ahinkorah, Sajjad Ahmad, Nasir Amanat, Dhanalakshmi Angappan, Jalal Arabloo, Armin Aryannejad, Seyyed Shamsadin Athari, Alok Atreya, Sina Azadnajafabad, Ahmed Y Azzam, Hassan Babamohamadi, Palash Chandra Banik, Mainak Bardhan, Alemshet Yirga Berhie, Ajay Nagesh Bhat, Julie Brown, Periklis Charalampous, Isaac Sunday Chukwu, Omid Dadras, Xiaochen Dai, Lalit Dandona, Rakhi Dandona, Fikadu Nugusu Dessalegn, Abebaw Alemayehu Desta, Nancy Diao, Daniel Diaz, Mahmoud Dibas, Deepa Dongarwar, Haneil Larson Dsouza, Michael Ekholuenetale, Nevine El Nahas, Muhammed Elhadi, Sharareh Eskandarieh, Adeniyi Francis Fagbamigbe, Jawad Fares, Ali Fatehizadeh, Seyed-Mohammad Fereshtehnejad, Florian Fischer, Richard Charles Franklin, Tushar Garg, Melaku Getachew, Fariborz Ghaffarpasand, Ali Gholamrezanezhad, Milad Gholizadeh Mesgarha, Sherief Ghozy, Mahaveer Golechha, Simon Matthew Graham, Vivek Kumar Gupta, Juanita A. Haagsma, Samer Hamidi, Netanja I. Harlianto, Mehdi Harorani, Mohammad Hasanian, Mohammed Bheser Hassen, Amir Human Hoveidaei, Farideh Iravanpour, Rana Irilouzadian, Chidozie C D Iwu, Louis Jacob, Chinwe Juliana Jaja, Charity Ehimwenma Joshua, Jacek Jerzy Jozwiak, Vidya Kadashetti, Amit Kandel, Rami S. Kantar, Ibraheem M Karaye, Samad Karkhah, Yousef Saleh Khader, Ejaz Ahmad Khan, Md Jobair Khan, Mohammad Saeid Khonji, Moein Khormali, Grace Kim, Vijay Krishnamoorthy, Senthil D Kumaran, Mohammad-Reza Malekpour, Mohamed Kamal Mesregah, Ana Carolina Micheletti Gomide Nogueira de Sá, Alireza Mirahmadi, Seyed Peyman Mirghaderi, Moonis Mirza, Awoke Misganaw, Sanjeev Misra, Yousef Mohammad,

Esmaeil Mohammadi, Ali H Mokdad, Holger Möller, Sara Momtazmanesh, Mohammad Ali Moni, Ebrahim Mostafavi, Francesk Mulita, Mohsen Naghavi, Hasan Nassereldine, Zuhair S Natto, Van Thanh Nguyen, Antonio Tolentino Nogueira de Sá, Andrew T Olagunju, Isaac Iyinoluwa Olufadewa, Abiodun Olusola Omotayo, Mayowa O Owolabi, Shankargouda Patil, Shrikant Pawar, Paolo Pedersini, Ionela-Roxana Petcu, Ali Mohammad Pourbagher-Shahri, Maryam Faiz Qureshi, Pankaja Raghav Raghav, Vafa Rahimi-Movaghar, Mosiur Rahman, Niloufar Rahnavard, Ali Rajabpour-Sanati, Mohammad-Mahdi Rashidi, Salman Rawaf, Nicholas L S Roberts, Basema Saddik, Umar Saeed, Mahdi Safdarian, Sara Samadzadeh, Abdallah M Samy, Arash Sarveazad, Mahan Shafie, Ataollah Shahbandi, Mequannent Melaku Sharew Sharew, Rahim Ali Sheikhi, Parnian Shobeiri, Sina Shool, Seyed Afshin Shorofi, Migbar Mekonnen Sibhat, Ehsan Sinaei, Paramdeep Singh, Surjit Singh, Yonatan Solomon, Houman Sotoudeh, Belsti Atnkut Tadesse, Aljoscha Thomschewski, Muhammad Umair, Sahel Valadan Tahbaz, Pascual R Valdez, Narayanaswamy Venketasubramanian, Nuwan Darshana Wickramasinghe, Ai-Min Wu, Fereshteh Yazdanpanah, Arzu Yigit, Zhi-Jiang Zhang.

Drafting the work or revising it critically for important intellectual content

Amirali Aali, Foad Abd-Allah, Aidin Abedi, Denberu Eshetie Adane, Saira Afzal, Bright Opoku Ahinkorah, Nasir Amanat, Jalal Arabloo, Seyyed Shamsadin Athari, Alok Atreya, Sina Azadnajafabad, Ahmed Y Azzam, Mainak Bardhan, Azadeh Bashiri, Ajay Nagesh Bhat, Julie Brown, Ana Paula Champs, Fikadu Nugusu Dessalegn, Nancy Diao, Daniel Diaz, Mahmoud Dibas, Deepa Dongarwar, Haneil Larson Dsouza, Muhammed Elhadi, Sharareh Eskandarieh, Adeniyi Francis Fagbamigbe, Jawad Fares, Ali Fatehizadeh, Seyed-Mohammad Fereshtehnejad, Florian Fischer, Tushar Garg, Melaku Getachew, Sherief Ghozy, Vivek Kumar Gupta, Juanita A. Haagsma, Netanja I. Harlianto, Amr Hassan, Amir Human Hoveidaei, Farideh Iravanpour, Rana Irilouzadian, Chidozie C D Iwu, Louis Jacob, Chinwe Juliana Jaja, Nitin Joseph, Jacek Jerzy Jozwiak, Vidya Kadashetti, Amit Kandel, Rami S. Kantar, Samad Karkhah, Yousef Saleh Khader, Ejaz Ahmad Khan, Md Jobair Khan, Hamid Reza Khayat Kashani, Grace Kim, Senthil D Kumaran, Mohammad-Reza Malekpour, Tuomo J Meretoja, Mohamed Kamal Mesregah, Tomislav Mestrovic, Ana Carolina Micheletti Gomide Nogueira de Sá, Alireza Mirahmadi, Seyed Peyman Mirghaderi, Moonis Mirza, Awoke Misganaw, Yousef Mohammad, Esmaeil Mohammadi, Ali H Mokdad, Holger Möller, Sara Momtazmanesh, Mohammad Ali Moni, Ebrahim Mostafavi, Mohsen Naghavi, Hasan Nassereldine, Zuhair S Natto, Huong Lan Thi Nguyen, Van Thanh Nguyen, Andrew T Olagunju, Abiodun Olusola Omotayo, Mayowa O Owolabi, Shankargouda Patil, Shrikant Pawar, Paolo Pedersini, Ionela-Roxana Petcu, Suzanne Polinder, Ali Mohammad Pourbagher-Shahri, Pankaja Raghav Raghav, Vafa Rahimi-Movaghar, Niloufar Rahnavard, Ali Rajabpour-Sanati, Salman Rawaf, Basema Saddik, Umar Saeed, Mahdi Safdarian, Sara Samadzadeh, Abdallah M Samy, Allen Seylani, Mahan Shafie, Pavanchand H Shetty, Parnian Shobeiri, Sina Shool, Seyed Afshin Shorofi, Migbar Mekonnen Sibhat, Paramdeep Singh, Yonatan Solomon, Aljoscha Thomschewski, Muhammad Umair, Sahel Valadan Tahbaz, Narayanaswamy Venketasubramanian, Linh Gia Vu, Nuwan Darshana Wickramasinghe, Ai-Min Wu, Arzu Yigit, Iman Zare.

Managing the estimation or publications process

Nasir Amanat, Ahmed Y Azzam, Fikadu Nugusu Dessalegn, Ali Fatehizadeh, Melaku Getachew, Farideh Iravanpour, Rana Irilouzadian, Samad Karkhah, Ali H Mokdad, Mohsen Naghavi, Van Thanh Nguyen, Mahdi Safdarian, Abdallah M Samy, Ai-Min Wu.
